# Immunoinformatics design of a multi-epitope vaccine for *Chlamydia trachomatis* major outer membrane proteins

**DOI:** 10.1038/s41598-024-81736-w

**Published:** 2024-12-02

**Authors:** Seema Shetty, Swagatika Dash, Avinash Kumar, Shashidhar Vishwanath, Suvarna G. Kini, Angela Brand

**Affiliations:** 1 Department of Microbiology, Kasturba Medical College, Manipal,, Manipal Academy of Higher Education, Madhav Nagar, Manipal, Karnataka 576104 India; 2 Faculty of Health, Medicine and Life Sciences, Maastricht University, 6229 GT Maastricht, The Netherlands; 3 Department of Pharmaceutical Chemistry, Manipal College of Pharmaceutical Sciences, Manipal Academy of Higher Education, Manipal, Karnataka 576104 India; 4 Department of Medical Affairs, Curie Sciences Private Limited, Samastipur, Bihar 848125 India; 5 Prasanna School of Public Health, Manipal Mc Gill Centre for Infectious Diseases, Manipal Academy of Higher Education, Manipal, Karnataka 576104 India; 6 Prasanna School of Public Health, Manipal Academy of Higher Education, Manipal, India; 7 United Nations University Maastricht Economics and Social Research Institute On Innovation and Technology (UNU-MERIT), 6211 AX Maastricht, The Netherlands

**Keywords:** Multi-epitope vaccine, *Chlamydia trachomatis*, Immunoinformatics, T-cell and B-cell epitopes, Molecular dynamics simulations, Immune simulation, Computational biology and bioinformatics, Immunology

## Abstract

*Chlamydia trachomatis* (CT) remains a significant infectious cause of blindness and sexually transmitted infections worldwide. The objective and novelty of this study lie in using different serovars of CT to design a broad-spectrum multi-epitope vaccine that might confer immunity against different CT infections. As the major outer membrane protein in CT has good immunodominance properties and high conservation and also determines the several serotypes of CT, it is selected as an antibody target in this study. T-cell and B-cell epitopes from serovars A, B, D, E, L1, and L2 were predicted and combined into a single construct by incorporating adjuvants and linkers to enhance immunogenicity and stability. Physicochemical characterization confirmed the constructed vaccine’s anti-allergic, immunogenicity, and thermostable characteristics, followed by structural modeling to refine its 3D configuration. The 3D model structure of the vaccine was validated through the Ramachandran plot and ProSA z-score. Molecular docking studies of the vaccine demonstrated stable binding with toll-like receptor 3, along with molecular dynamics simulations and binding free energy calculations supporting the complex’s stability. In silico cloning has indicated a high potential for expression in *Escherichia coli*. Lastly, immune simulations revealed robust activation of B cells, cytotoxic T cells, and antigen-presenting cells, alongside significant production of IgM, IgG antibodies, and balanced Th1/Th2 cytokine response, which is crucial for effective immunity. These results suggest the multi-epitope vaccine could effectively induce comprehensive immune responses against CT, highlighting the need for further in vivo validation to advance this promising candidate toward clinical use.

## Introduction

*Chlamydia trachomatis*(CT) is a gram-negative bacterium whose only natural host is humans. These obligate intracellular pathogenic bacteria are significant etiological agents of several sexually transmitted diseases (STDs) and preventable blindness, known as trachoma. In 2020, approximately 128.5 million new cases of chlamydia infection were reported globally^1^. Similarly, an estimated 125 million people were affected by high-risk trachoma in 2022^2^. CT strains comprise three biovars depending on the pathological conditions they cause in humans. These biovars are classified into various serovars according to the characteristics of surface antigens, major outer membrane protein (MOMP)^3^. The trachoma biovar caused by serovars A-C is the world’s major infectious cause of blindness and visual impairment. Serovars D-K infects the genital tract, causing urethritis, cervicitis, and pelvic inflammatory disease (PID) in females. The severe form of this infection also causes miscarriage, preterm delivery, ectopic pregnancy, and infertility. The severe other consequence caused by serovars D-K is epididymitis, resulting in infertility in males. Serovars L1 to L3 cause invasive infections in the lymphatic system named lymphogranuloma venereum^4^. Genital tract CT infections are also linked to a relatively more significant risk of acquiring cervical carcinoma and infection with the Human Immunodeficiency Virus (HIV)^5^. The prospective severity of CT pathology emphasizes its relevance as an important public health concern worldwide and requires effective disease management and prophylaxis.

*Chlamydia trachomatis*has a biphasic developmental form: the infectious form termed elementary bodies (EBs), and the noninfectious form termed reticulate bodies (RBs). The infection starts after the attachment of EBs with epithelial cells of the urogenital tract and then differentiates through binary fission to RBs for further multiplication. After replication, the RBs again transform back to EBs, expelled from the cell to infect new neighboring cells^6^. The transmission of *Chlamydia trachomatis*generally occurs from the genitourinary system to the eyes and vice versa. CT genital infections are known to be sexually transmitted but can also transmit vertically from an infected pregnant female to the fetus and during childbirth by infected vaginal delivery. This infection also spreads through contaminated fingers and towels like other fomites in populations with poor hygiene^7^. Chlamydial infections remain asymptomatic and show fewer symptoms than other STDs, so the pathogens remain untreated till the secondary or tertiary stages of the infections^8^. As CT bacteria are susceptible and responsive to antibiotics such as macrolides, tetracyclines, fluoroquinolones, rifampin, and some beta-lactam antibiotics, clinical treatment failures are usually reported due to developing heterotypic resistance in *C. trachomatis*^9^. This resistance may arise because of steady growth in a particular environment or stress response obstructing the bacteria from antibiotic treatment^10^. Due to the asymptomatic nature of CT and inappropriate treatment compliance, the infections cause severe reproductive consequences. Therefore, an adequate immune response is highly demanded to reduce the infections caused by Chlamydia. This protective immunity is associated with the production of IFN-γ cytokine by CD4 + and CD8 + T-cells^11^. In this regard, developing the CT vaccine is a promising strategy to manage infections.

The initial step for developing a subunit vaccine is to choose virulent antigenic proteins, unlike the human host. Various highly conserved surface-associated proteins defining serotypes in *C. trachomatis*may be regarded as a prominent antigenic target for the vaccine. Among them, the cysteine-rich major outer membrane proteins (MOMP) are the most appropriate candidate for vaccine development. MOMP composes around 60% mass of the outer membrane protein and contains several immunodominant T- and B-cell epitopes that induce distinct immune responses against Chlamydia. These MOMPs transport sugars, ions, and nucleotides across the membrane and also help CT during adhering to the host cells^12^. The flexible loops present in the extracellular surface of CT, known as variable domains (VD), mainly show antibody responses and have attracted interest in vaccine development^13^. The leading vaccine candidate for CT infections, CTH522, formulated with CAF®01 adjuvant, is also composed of using MOMP VD4 regions of serovars (D, E, F, and G) and has now passed Phase I clinical trials^14^. Presently, in-silico immunoinformatic techniques with a reverse vaccinology approach have been used for muti-epitope vaccine design to facilitate developmental time at a minimum cost^15^. Thus, in this regard, we tried to design a new multi-epitope vaccine against *C. trachomatis* infections by targeting the MOMP of serovars (A, B, D, E, L1, and L2) through several bioinformatics tools.

In this research, first, the protein sequences of the selected serovars were retrieved and used for the prediction of CTL, HTL, and B-cell epitopes using IEDB and ABCpred servers. The vaccine was constructed by joining the selected epitopes through appropriate linkers to build the main structure of the vaccine. The 50S ribosomal protein L7/L12 (Locus RL7_MYCTU) as an adjuvant was attached at the initial N-terminal to overcome the poor immunogenicity issue. The physicochemical parameters, allergenicity, antigenicity, and toxicity of the constructed vaccine were evaluated. Next, the 2D and 3D structures of the vaccine construct were predicted. The selected 3D structure was taken forward to investigate the binding ability of the vaccine with the immune receptor of TLR3 through several methods, such as molecular docking, followed by binding energy estimation and molecular dynamics (MD) simulations. Finally, in silico cloning and immune simulations of the designed vaccine were performed to evaluate immune response. An attempt to design a novel multi-epitope vaccine is made in this study, which may assist in developing a new vaccine against *C. trachomatis* infections in the future.

## Materials and methods

### Retrieval of MOMP amino acid sequence

For this study, the protein sequence of several antigenic variants of MOMP or serovariants of ocular tropic strains (A, B), urogenital tropic strains (D, E), Lymphogranuloma venereum strains (L1, L2) of *C. trachomatis* has been selected. The amino acid sequences were retrieved from the UniProt database (https://www.uniprot.org/) in the FASTA format^16^.

### Cytotoxic T-lymphocyte (CTL) epitopes prediction

The CD8^+^T-cells are MHC-I confined and a part of white blood cells (WBCs)^17^. IEDB MHC I binding (http://tools.iedb.org/mhci/) sever was utilized to assess the binding ability of a protein to CTL^18^. NetMHCpan 4.1 EL (recommended epitope predictor-2023.09) prediction method with humans as MHC source species was selected for the analysis. From the human leukocyte antigen (HLA) allele reference set, five different alleles such as HLA-A*02:01, HLA-A*02:06, HLA-A*24:02, HLA-B*40:01, and HLA-B*40:02 were chosen. The epitope chain length was set to 9. The resulting output in predicted scores was considered for sorting the peptide sequences.

### Helper T-lymphocyte (HTL) epitopes prediction

The CD4^+^T-cells are activated after binding of antigen MHC-II complex (HTL)^19^. The predictions of antigen-specific immune response or HTL for each serovar have been carried out using IEDB MHC II binding (http://tools.iedb.org/mhcii/) with NetMHCIIpan 4.1 EL (recommended epitope predictor-2023.09) prediction method^20^. The species/locus was selected as Human/HLA-DR with a 15-mer epitope length option. Next, we selected four different sets of alleles, namely HLA-DRB1*01:15, HLA-DRB1*08:02, HLA-DRB1*12:01, and HLA-DQA1*01:07/ DQB1*05:03 from 7-allele HLA (human leukocyte antigen) reference set. The predicted results were interpreted as percentile score and rank.

### Linear B-cell epitopes prediction

ABCpred server (http://www.imtech.res.in/raghava/abcpred/) has been utilized in this study to predict the linear B-cell epitopes^21,22^. For this study, the epitope length of 16 and 0.51 threshold was fixed for prediction analysis.

### Screening and characterization of epitopes

All the epitopes from different serovars were pooled, and only the best epitopes from individual serovars were considered for constructing the CT vaccine candidate. The best epitopes were selected based on score and percentile rank. The antigenic property related to the immune response must not be the only criteria for selecting epitopes, but the epitopes should be devoid of allergens and toxins. As a result, the antigenicity, allergenicity, and toxicity characteristics of the chosen epitopes were predicted using the VaxiJen v2.0 server (https://www.ddg-pharmfac.net/vaxijen/VaxiJen/VaxiJen.html), AllergenFP v.1.0 server (https://www.ddg-pharmfac.net/AllergenFP/), and ToxinPred server (https://webs.iiitd.edu.in/raghava/toxinpred/), respectively^23^.

VaxiJen v2.0 is the first-ever server based on an alignment-independent approach for antigenicity prediction. It uses the physicochemical characteristics of proteins without relying on sequence alignment. The accuracy of this server’s performance ranges from 70 to 89%, depending on the model organism used^24,25^. The AllerFP v.1.0 server differentiates allergen from non-allergen using hydrophobicity and β-strand forming tendency of amino acids through the fingerprinting approach^26^. The ToxinPred server predicts the toxicity of the peptides as well as the crucial physical and chemical properties of the peptide sequence, such as hydropathicity, hydrophobicity, hydrophilicity, molecular weight, and p^I^charge^27,28^.

### Molecular docking of selected epitopes with HLA alleles

To gain an insight into how CTL epitopes interact with various HLA molecules, we carried out molecular docking studies of all six selected CTL epitopes with HLA-A*02:01 (PDB ID:4U6Y), HLA-A*02:06 (PDB ID: 3OXR), HLA-A*24:02 (PDB ID: 8SBK), and HLA-B*40:02 (PDB ID:5IEH). For docking these epitopes, the HPEPDOCK 2.0 (http://huanglab.phys.hust.edu.cn/hpepdock/) webserver was used, which performs a flexible peptide-protein docking by fast modeling of peptide conformations and global/local sampling of binding orientations^29^.

### Multi-epitope vaccine construction and its conservancy analysis

The multi-epitope vaccine structure was constructed by linking the screened epitopes (T and B-cell) and adjuvants with appropriate linkers. First, the 50S ribosomal protein L7/L12 (Locus RL7_MYCTU) was introduced as an adjuvant (Accession no. P9WHE3) at the N-terminal ends by employing EAAAK linker to enhance the immunogenicity of the constructed vaccine^30^. The adjuvant sequence was obtained from the UniProt database. Like natural linkers, this EAAAK linker exhibited an alpha helix structure and functions as a rigid spacer to enhance stability and maintain a specific distance for the independent functioning of domains^31^. AAY (Ala-Ala-Tyr) linker joined the selected T-cell epitopes (CTL and HTL), while KK (the lysine) linker joined the selected B-cell epitopes. The AAY linker, identified as the cleavage site of the mammalian proteasome, is mainly used to reduce junctional immunogenicity and improve pathogen-specific immunity^32,33^. The KK linker is the lysosomal protease (cathepsin B) target sequence, which is one of the crucial proteases required for antigen processing in the area of MHC-II antigen presentation. The joining of two peptides through the KK linker can expose each peptide to antibodies by preventing the induction of antibodies to the amino acid sequence, which is formed by combining two peptides^34^. A 6x-His tail for facilitating protein purification and identification was attached at the C-terminal end to construct the final vaccine structure^35^. The conservancy analysis of the final constructed vaccine was carried out without taking the adjuvant across all the peptide sequences of the selected serovars of *C. trachomatis*^36^.

### Evaluation of physicochemical features, antigenicity, allergenicity, and toxicity of constructed vaccine

Physicochemical properties analysis helps guide the constructed vaccine’s *in vitro* and *in vivo*evaluation^37^. So different physicochemical parameters of the vaccine design, including number of amino acids, molecular weight, amino acid composition, theoretical p^I^, atomic formula and composition, extinction coefficient, estimated half-life, aliphatic index, instability index, and grand average of hydropathicity (GRAVY) were characterized using ProtParam online tool (https://web.expasy.org/protparam/)^38^. Protein-Sol (https://protein-sol.manchester.ac.uk/), a web server, was employed to analyze the solubility of the designed vaccine^39^.

To predict antigenicity of the structured vaccine, ANTIGENpro (https://scratch.proteomics.ics.uci.edu/) and VaxiJen v2.0 (https://www.ddg-pharmfac.net/vaxijen/VaxiJen/VaxiJen.html) were employed^40^.

The allergic properties of the query vaccine structure were predicted employing AllerTOP v.2.0 (https://www.ddg-pharmfac.net/AllerTOP/) and AllergenFP v.1.0 (https://www.ddg-pharmfac.net/AllergenFP/) servers^41^. The constructed vaccine was determined as toxic or non-toxic by the ToxinPred web server (https://webs.iiitd.edu.in/raghava/toxinpred/)^27^.

### Vaccine secondary structure prediction

The secondary structure of the vaccine sequence was predicted by using different online servers, including PSIPRED (http://bioinf.cs.ucl.ac.uk/psipred/), Prabi (https://npsa-prabi.ibcp.fr/cgi-bin/npsa_automat.pl?page=/NPSA/npsa_gor4.html) and RaptorX (http://raptorx6.uchicago.edu/)^42,43^. The prediction of solvent accessibility (ACC), disorder regions (DISO), and 3-state secondary structure of the protein was carried out employing the RaptorX server^44^.

### Vaccine 3D structure modeling, refinement, and validation

The tertiary structure of the vaccine design was modeled to explore the binding interactions of the constructed vaccine with innate immune receptors. I-TASSER software (https://zhanggroup.org/I-TASSER/) performed the modelling procedure^45,46^. The models were downloaded in PDB format from I-TASSER and were visualized by Schrodinger Drug Discovery Suite (Maestro Version 11.8) for the production of figures. The best homology model selected from the I-TASSER server was further optimized by refining the method. First, ModRefiner (https://zhanggroup.org/ModRefiner/) and then GalaxyWEB Refine (https://galaxy.seoklab.org/cgi-bin/help.cgi?key=METHOD&type=REFINE) web servers were used for refinement protocol^47,48^. The GalaxyWEB Refine server determined the results by the GDT-HA score, root means square deviation (RMSD) value, MolProbity score, Clash score, and Rama favoured values^49^.

Model validation is a crucial step for detecting probable errors in 3D structures and comparing refined models’ quality before and after the refining process^50^. For this evaluation, ProSA-web (https://prosa.services.came.sbg.ac.at/prosa.php) and SAVES v6.0 (https://saves.mbi.ucla.edu/) servers were utilized. It plots the calculated Z-score of the developed model with Z-scores of already developed protein 3D structures available in PDB^51^. Further, the developed vaccine model was substantiated with the SAVES v6.0 server. This server runs several programs like ERRAT, Verify3D, and PROCHECK^52,53^. Ramachandran plot was obtained from the PROCHECK application^54,55^.

### Vaccine candidate disulfide engineering for stability

The formation of new disulfide bonds can be achieved by mutations in the other amino acids to cysteine^56^. In this study, we predicted the amino acid pairs capable of being substituted by cysteine to generate disulfide bonds via the Disulfide by Design v 2.0 web server (http://cptweb.cpt.wayne.edu/DbD2/)^57^.

### Prediction of discontinuous B-cell epitopes

The 3D structure of the constructed vaccine was used to predict the discontinuous B-cell epitopes through the ElliPro (http://tools.iedb.org/ellipro/) web tool. The program was run with default epitope prediction parameters (minimum score of 0.5 and maximum distance of 6 Å) present on the website^58^.

### Prediction of cytokines-inducing potential

IFN-γ inducing potential of the designed vaccine has been predicted via the IFNepitope server (https://webs.iiitd.edu.in/raghava/ifnepitope/predict.php)^59,60^.

### Molecular docking studies and binding energy estimation

Protein-protein docking was carried out against TLR3 (PDB:1ZIW) receptor. The binding sites of both proteins were predicted employing the CASTp server (http://sts.bioe.uic.edu/castp/index.html?1bxw). This server mainly predicts the functional regions and surface features of the protein^61^. Online docking webserver GRAMM-X (https://gramm.compbio.ku.edu/) was utilized to perform molecular docking. LIGPLOT v.4.5.3 was employed to visualize the interactions of TLR3 with the vaccine. The free binding energy of the bounded structures was calculated using the PRODIGY webserver to choose the top-ranked docking result. Finally, the docked complex with the lowest free binding energy value was considered for further molecular dynamics (MD) simulations.

### Molecular dynamics simulation and binding free energy analysis

MD simulation was conducted using GROMACS (version 2018.3). The AMBER ff99SBILDN force field and TIP3P solvent model were utilized. Ligand topology was created with the Antechamber tool from AMBERTOOLS. The solvated complex underwent energy minimization through the conjugate gradient and steepest descent (SD) algorithms. After minimization, a two-phase equilibration was carried out, followed by a 100 ns MD simulation at 300 K and 1 bar with a two fs time step^62^. Following the MD simulations, an MM-PBSA study was conducted to calculate the binding free energies of the chosen docked complex using the g_mmpbsa tool^63^.

### In silico cloning and codon adaptation

The peptide sequence of the vaccine was subjected to VectorBuilder online software (https://en.vectorbuilder.com/tool/codon-optimization.html)^64^. For codon adaptation, *E.coli* str. K-12 substrate. MG1655 was opted as a host to express the final vaccine. The optimized sequence was put in the SnapGene tool (https://www.snapgene.com/) by introducing two restriction sites (MlsI and AdeI) at the C- and N-terminals of the vaccine sequences. In the final step, the optimized vaccine sequence and the incorporated restriction sites were inserted into the pPIC6 A vector employing the SnapGene tools for *in silico* cloning.

### Immune simulation

The designed vaccine’s real-life-like immunogenicity and immune response evaluation was performed through computational immune simulation using C-IMMSIM (https://kraken.iac.rm.cnr.it/C-IMMSIM/index.php) server^65^.

## Results

### Prediction, characterization, and selection of T-cell and B-cell epitopes

The interaction between antigen and antibody is critical in vaccine research. Antibodies attach to the desired antigen molecule in a particular pattern or on the part of the antigenic structure known as an epitope for developing immunity. T-cell and B-cell epitopes significantly build immune responses against various infectious diseases. T-cell epitope prediction aims to find peptides with the smallest sequence length inside an antigen that may stimulate CD4^+^ or CD8^+^T-cells. T-cells can recognize the antigenic peptides through the binding pattern of MHC-I or MHC-II molecules^66^. The IEDB server predicted both CTL and HTL epitopes for each serovar. Using the ABCpred server, the B-cell epitopes for each serovar were predicted. The epitopes were selected based on score and percentile rank. All the epitopes from different serovars were further evaluated for antigenicity, allergenicity, and toxicity. The results are provided in the **Supplementary Table S1**. After all the evaluations, the best epitopes from serovars A, B, D, E, L1, and L2 were considered for constructing the CT vaccine candidate. For this, six epitopes from CTL (9-mer), HTL (15-mer), and B-cell (16-mer) epitopes were chosen (Table 1).

**Table 1:** List of T-cell (CTL, HTL) and B-cell epitopes selected from MOMP of different serovars for vaccine candidate construction.

Serovars	CTL (9-mer) epitope	HTL (15-mer) epitope	B-cell (16-mer) epitope
A	HEWQASLAL	ETRLIDERAAHVNAQ	TGNATAPTTLTARENP
B	SLDQSVVEL	TMQIVSLQLNKMKSR	TGNAVAPSTLTARENP
D	AESVPNMSF	DTMQIVSLQLNKMKS	EGFGGDPCDPCATWCD
E	LYTDTAFSW	ADTIRIAQPKSATAI	EGFGGDPCDPCTTWCD
L1	FVFDRVLQT	NKEFQMGAKPTATTG	TGTKDASIDYHEWQAS
L2	LYTDTTFAW	ADTIRIAQPKSATTV	AQPKSATTVFDVTTLN

### Molecular docking analysis of selected epitopes with HLA alleles

The selected CTL epitopes were subjected to molecular docking with HLA molecules using the HPEPDOCK 2.0 server to verify their interaction with HLA alleles. The selected CTL epitopes with HLA molecules showed favorable binding poses. The docking scores of each selected CTL epitope ranged from −130 to −261 kcal/mol, suggesting that the selected CTL epitopes would bind with HLA molecules. The detailed docking results of CTL epitopes and HLA molecules are given in Table 2. The 3D binding interactions of CTL epitopes with various HLA alleles were generated and provided in **Supplementary Figures S1-S24**.

**Table 2:** Molecular docking scores of CTL epitopes with different HLA molecules.

Serovars	Epitope	HLA type	PDB	Docking score (Kcal/mole)
A	HEWQASLAL	HLA-A*02:01	4U6Y	−261.284
		HLA-A*24:02	8SBK	−174.133
		HLA A*02:06	3OXR	−195.890
		HLA-B*40:02	5IEH	−233.564
B	SLDQSVVEL	HLA-A*02:01	4U6Y	−133.406
		HLA-A*24:02	8SBK	−138.005
		HLA A*02:06	3OXR	−145.426
		HLA-B*40:02	5IEH	−132.083
D	AESVPNMSF	HLA-A*02:01	4U6Y	−194.271
		HLA-A*24:02	8SBK	−161.007
		HLA A*02:06	3OXR	−173.940
		HLA-B*40:02	5IEH	−175.773
E	LYTDTAFSW	HLA-A*02:01	4U6Y	−188.580
		HLA-A*24:02	8SBK	−241.453
		HLA A*02:06	3OXR	−194.811
		HLA-B*40:02	5IEH	−195.831
L1	FVFDRVLQT	HLA-A*02:01	4U6Y	−185.867
		HLA-A*24:02	8SBK	−192.325
		HLA A*02:06	3OXR	−222.287
		HLA-B*40:02	5IEH	−196.032
L2	LYTDTTFAW	HLA-A*02:01	4U6Y	−204.330
		HLA-A*24:02	8SBK	−199.181
		HLA A*02:06	3OXR	−188.802
		HLA-B*40:02	5IEH	−239.813

### Multi-epitope peptide construction and its conservancy analysis

**Fig. 1:**
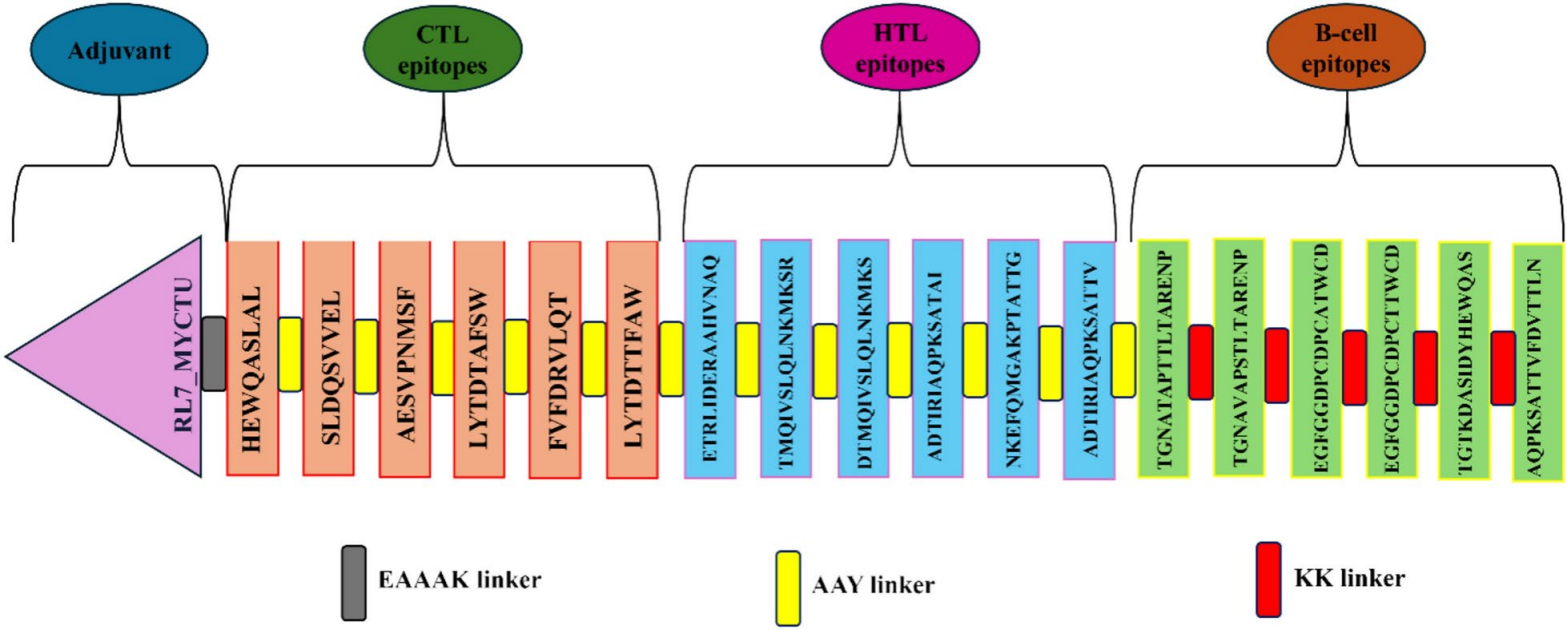
Schematic visualization of the multi-epitope vaccine structure containing 427 amino acids.

Peptide vaccines have become good alternatives to protein- and organism-based vaccines because they are more precise, more specific in showing actions, and easy to prepare at a low cost. The peptide-based vaccines have poor immunogenic responses but can becountered by fusing numerous epitopes^67^. The final muti-epitope vaccine was comprised of five parts: immunoadjuvant of a 50S ribosomal protein L7/L12, 6 CTL epitopes, 6 HTL epitopes, 6 B-cell (linear) epitopes, and a 6xHis tail. The adjuvant was placed at the N-terminus end of the vaccine to provide stability. This adjuvant was connected to the CTL epitope through the EAAAK linker. This adjuvant acts as a Toll-like receptor (TLR) agonist to improve the immunogenicity of the vaccine. As mentioned in Table, the final set of selected CTL and HTL epitopes were joined via the AAY linker, while the KK linker was employed to fuse the selected B-cell epitopes. Additionally, a 6xHis tag was introduced at the C-terminus end to aid in the protein identification and purification process. The final designed vaccine candidate has 427 amino acids drawn from 18 merged epitopes, as depicted in Fig. 1. The constructed vaccine sequence was evaluated for conservancy analysis without including the adjuvant across all the peptide sequences of the selected serovars of CT. The results demonstrated a minimum and maximum conservancy of 65.41% across all the chosen serovariants of CT.

### Physicochemical characterization, antigenicity, and allergenicity analysis of constructed vaccine

According to the results of the biochemical characteristics of the vaccine construct by the Protparam server, 427 amino acids containing hypothetical protein had a molecular weight of 45.7 kDa (<110 kDa) with molecular formula C_2032_H_3184_N_538_O_635_S_14_. The value indicated the suitability of the protein for development because smaller proteins are more accessible to clone and express than larger proteins. The theoretical isoelectric point (p^I^) value was predicted as 5.59. The p^I^value can be used to select and optimize protein purification methods such as isoelectric focusing and ion-exchange chromatography^68^. The aliphatic index was computed to be 74.54 (>50), indicating this to be thermostable. The aliphatic index is the proportion of protein occupied by aliphatic side chains, which measures the thermostability properties of peptide^69^. The designed vaccine had an instability index value of 21.37 (<40), demonstrating its stability. The extinction coefficient values were 55,275 and 55350 M^−1^cm^−1^ when all Cysteine residues converted to cystines and after the reduction of all cysteine residues. The 3D vaccine model had 54 negatively charged residues (no of Asp + Glu) and 45 positively charged residues (no of Arg + Lys). The half-life was 30 hrs in mammalian reticulocytes (*in vitro*), >20 hrs in yeast (*in vivo*), and >10 hrs in E. coli (*in vivo*). The query sequence of the vaccine exhibited the GRAVY (Grand average of hydropathicity) score of −0.219, illustrating its amphipathic behaviour and strong interaction with water molecules^70^. The predicted solubility value of 0.496 (>0.45) computed by the Protein-sol web server demonstrated the vaccine tends to be soluble during overexpression in E. coli^39^.

The estimated antigenicity of the designed protein sequence (with adjuvant) was 0.946348; without adjuvant, it was 0.970691, calculated by the ANTIGENpro server. As per antigenicity prediction by VaxiJen v2.0, the antigenicity of the vaccine design, including the adjuvant sequence, was predicted to be 0.5104, and without the adjuvant, it showed the value of 0.5511 in a bacterial model with a threshold of 0.5. The vaccine sequence was also analyzed as probable non-allergen using AllerTOP v.2.0 & AllergenFP v.1.0 servers. The predicted vaccine sequence was non-toxic based on the ToxinPred result. All the physicochemical parameters of the designed vaccine sequence are depicted in Table 3.

**Table 3:** Table illustrating the physicochemical and immunological behaviours of the designed vaccine.

Physicochemical parameters	Results
Number of amino acids	427
Molecular weight	45,759.70
Formula	C_2032_H_3184_N_538_O_635_S_14_
Theoretical isoelectric point (p^I^)	5.59
Total number of atoms	6403
Aliphatic index	74.54
Instability index	21.37
Extinction coefficients (all pairs of Cys residues form cystines)	55,725
Extinction coefficients (all Cys residues are reduced)	55,350
Total number of negatively charged residues (Asp + Glu)	54
Total number of positively charged residues (Arg + Lys)	45
Estimated half-life	30 h (mammalian reticulocytes, in vitro) > 20 h (yeast, in vivo) > 10 h (*E. coli*, in vivo*)*
GRAVY	−0.219
Predicted scaled solubility	0.496
Antigenicity	0.946348/ Antigen
ANTIGENpro	
VaxiJen v2.0	0.5104/ Probable antigen
Allergenicity (AllerTOP v.2.0 & AllergenFP v.1.0)	Probable non-allergen
Toxicity (ToxinPred)	Non-toxic

### Secondary structure prediction

**Fig. 2:**
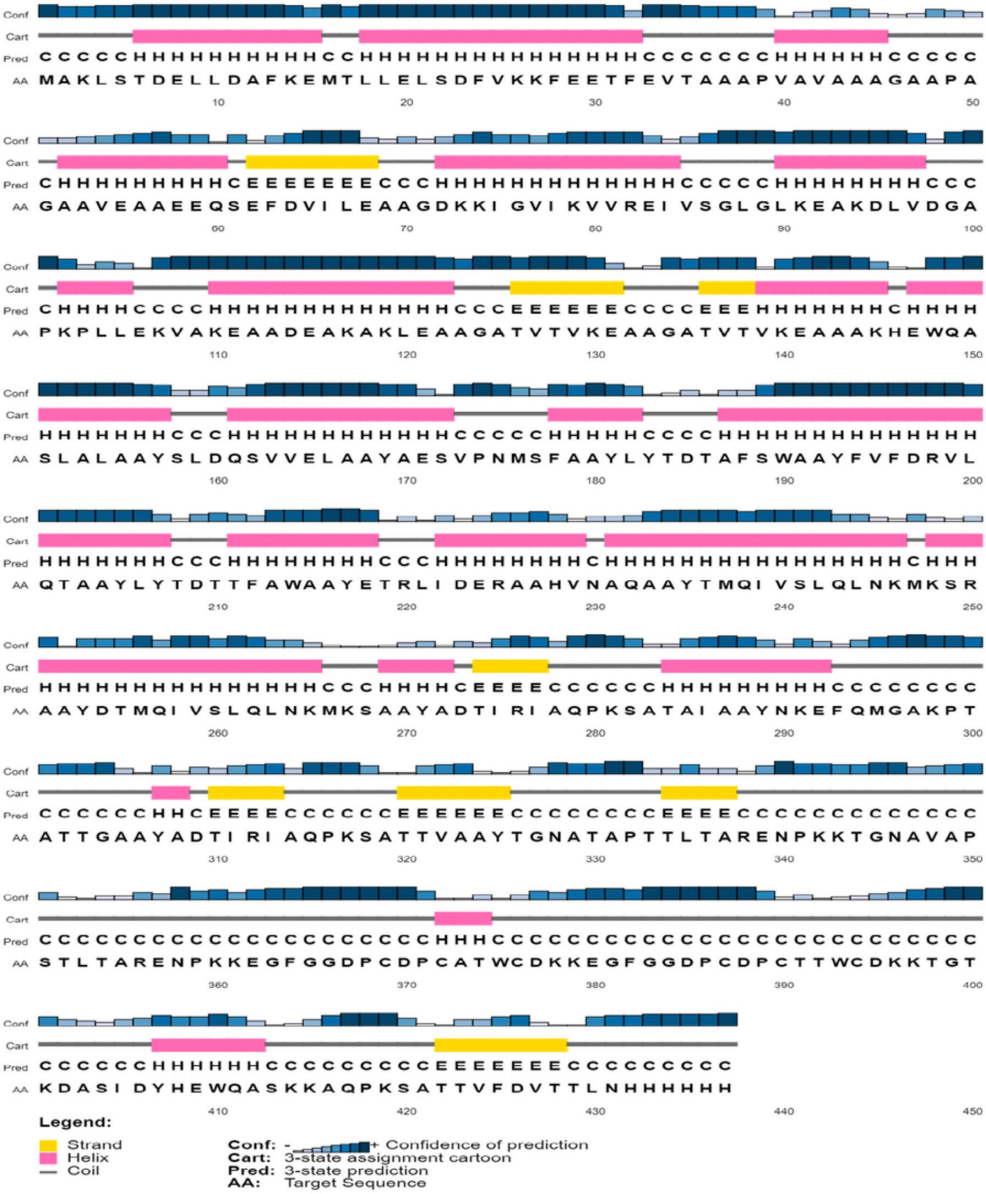
Secondary structure of the *C. trachomatis* vaccine sequence (displaying helix, strand, and coils) predicted by the PSIPRED.

**Fig. 3:**
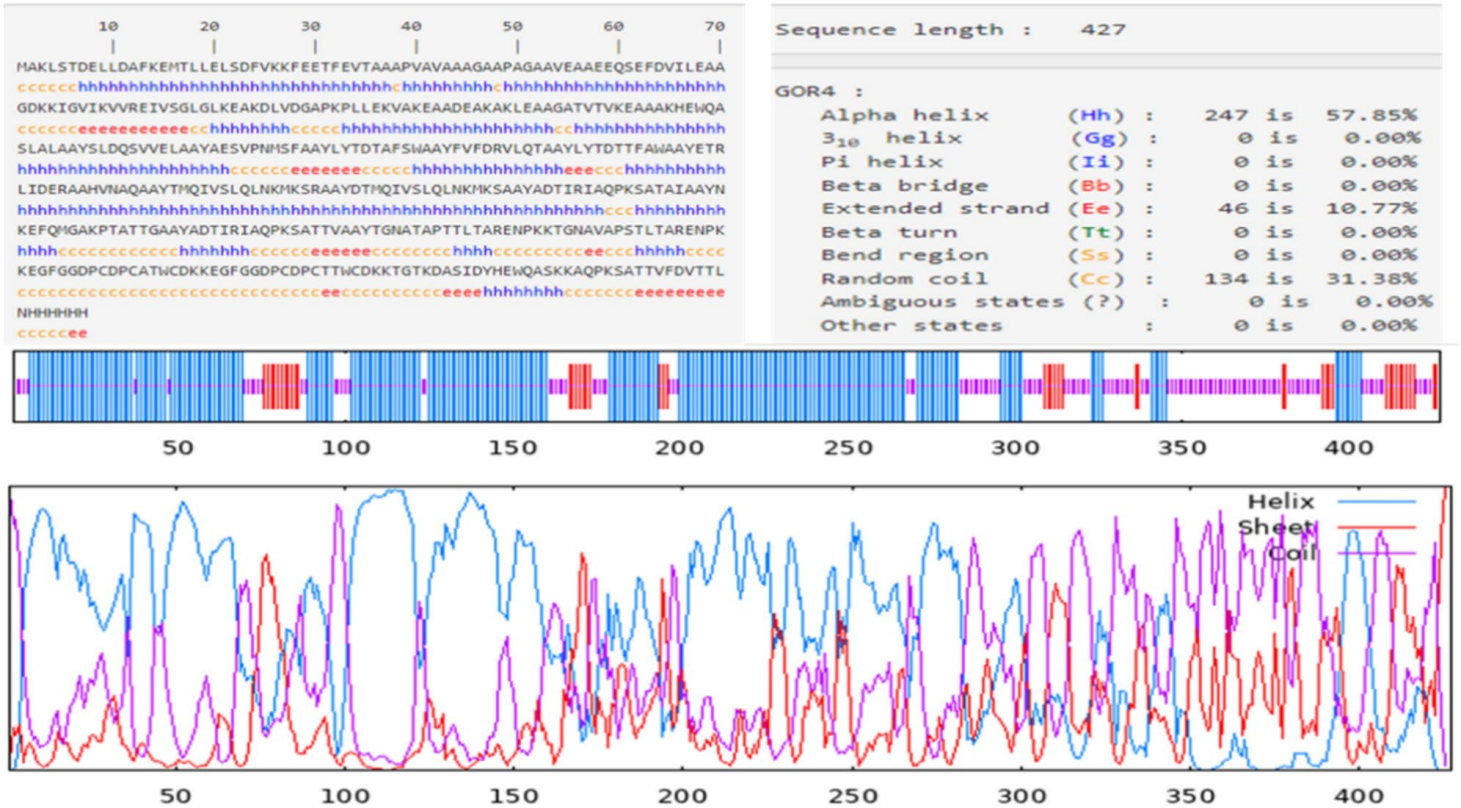
Representation of Prabi server predicted secondary structure of the vaccine construct.

The secondary structure of the final vaccine sequence was predicted by the PSIPRED web tool based on the physicochemical characteristics of the amino acid sequences, and it is depicted in Fig. 2. The results expected by the GOR4 method-based Prabi server revealed that the vaccine was constructed by 57.85% of α-helix, 10.77% of the extended strand, and 31.38% of the random coil (Fig. 3). The solvent accessibility of the query sequence, as estimated by the RaptorX online server, demonstrated that 51% of the total amino acids were exposed, 18% were medium-exposed, and 29% were buried. A total of 53 (12%) of residues were detected as disordered. This server also predicted a 3-state secondary structure of 36%, 13%, and 49% of α-helix, β-sheets, and coils, respectively.

### 3D structure prediction, refinement, and validation

The I-TASSER server (https://zhanggroup.org/I-TASSER/) has predicted the tertiary structure of the vaccine design. The top five 3D models were predicted using the best 10 threading templates with PDB hits 1rquA, 7soxA, 2ftc, 8ugcA, 8ej5A, and 8aucA. All the threading templates showed good alignment, as evidenced by Normalized Z-score values ranging from 0.92 to 5.66. The top one model was selected based on its C-score (−0.56), TM-score (0.64±0.13), and RMSD value (8.2±4.5Å). The C-score is otherwise known as the confidence score, indicating the quality of the model. The typical range of C-score varies from −5to 2, where a higher value reflects high confidence. TM-score and RMSD value are significant standards for identifying structural similarity and are mainly used for measuring the accuracy of structural modeling. However, the TM-score is more accurate than RMSD because local errors do not affect it^71^. The TM-score greater than 0.5 indicates the correct topology of the model, and its value in the selected model denotes the suitable topology^45^.

**Fig. 4:**
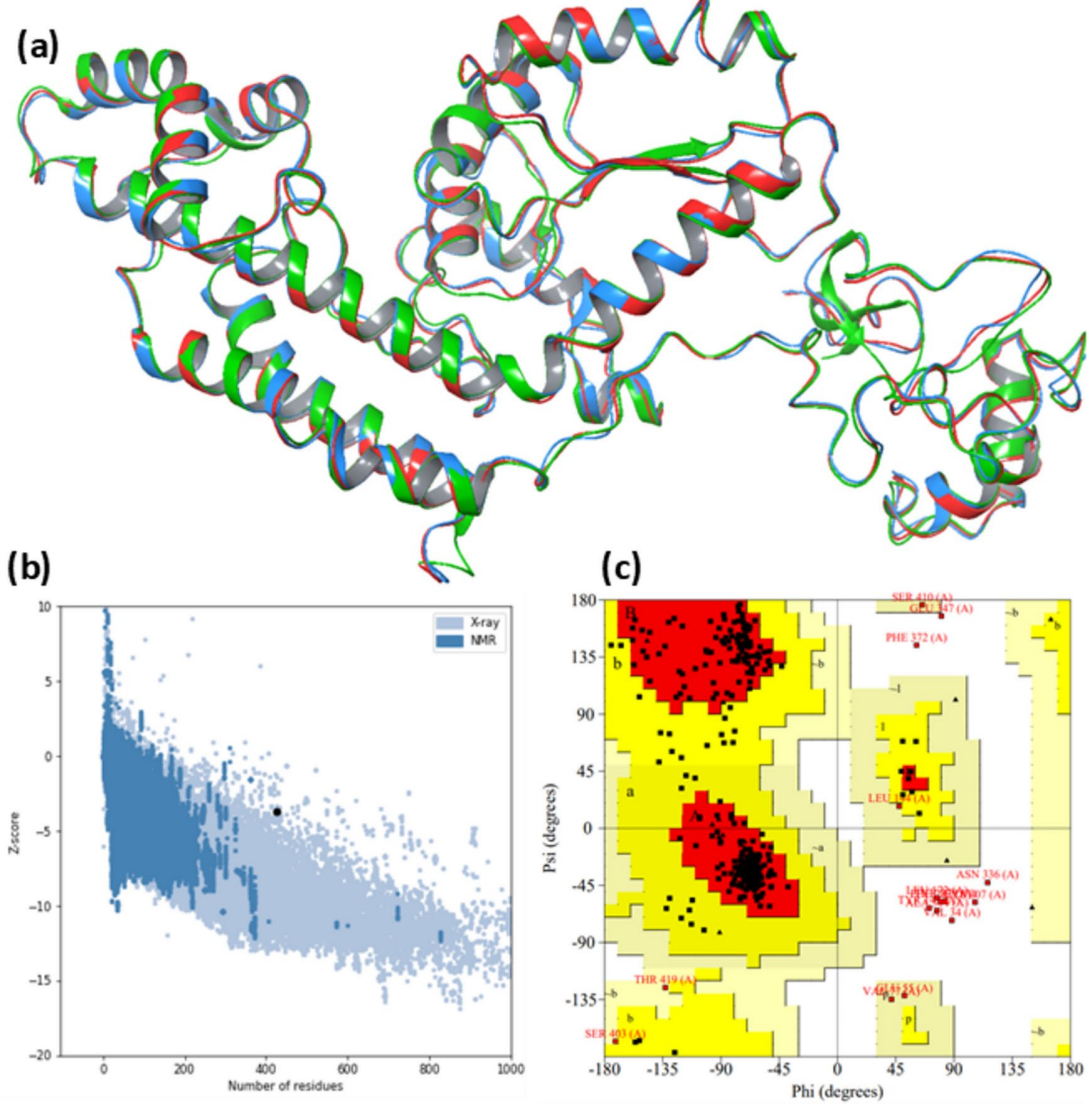
**(a)** The Red ribbon shows the original 3D structure of the proposed vaccine built by I-TASSER**;** the green ribbon shows the refined model of the vaccine using the ModRefiner server, and the blue ribbon shows the final three-dimensional model of the vaccine after refinement using the GalaxyRefine server.** (b)** Plot illustrating Z-score obtained by ProSA- web software. **(c)** PROCHECK Ramachandran plot of the selected vaccine 3D model.

The selected model has been further refined using ModRefiner and GlaxyRefine server. Five models were generated by the GalaxyRefine server (Table 4). And based on model quality scores such as GDT-HA score (0.9344), RMSD value (0.456), MolProbity score (2.113), clash score (11.3), poor rotamers (0.6) and Rama favoured (90.1), MODEL 5 was identified as the best model. All three models have been superimposed and shown in (Fig. 4a). For further confirmation and improvement of the accuracy of the refined model, we employed ProSA-web and SAVES v6.0 online server to validate the refined tertiary structure. The Z-scores for the initial model and all the refined models computed by ProSA-web are mentioned in Table 5, and the highest Z-score was found to be −3.68 for MODEL 5 (Fig. 4b). By the SAVES v6.0 online server, several programs like ERRAT, Verify3D, and PROCHECK were run to analyze the refined 3D structure. The ERRAT value demonstrates the resolution quality of the model by analyzing the non-bonded interactions occurring in different atoms. MODEL 5 had an ERRAT value of 83.249, higher than other refined models. It was also ascertained as compatible with its own building amino acid sequences as per calculation by Verify3D. Furthermore, the Ramachandran plot of the selected MODEL 5 was assessed by using the PROCHECK tool. This tool also investigates the stereochemical quality of the 3D atomic model by analyzing both residual and complete structural geometry. The Ramachandran plot demonstrated the Psi and Phi angles of 427 amino acids that prepare the 3D structure. The plot displayed 86.6% or 337 residues in Ramachandran’s favored area, with 42 residues (10.8%) in permitted regions and 2.6% or 10 residues of Ramachandran outliers, as shown in (Fig. 4c).

**Table 4:** Detailed result of model refinement by the GalaxyRefine server.

Model	GDT-HA	RMSD	MolProbity	Clash score	Poor rotamers	Rama favoured
Initial	1.0000	0.000	3.108	7.7	15.4	76.0
MODEL 1	0.9391	0.445	2.163	12.2	0.3	89.4
MODEL 2	0.9456	0.431	2.172	12.7	0.6	89.6
MODEL 3	0.9403	0.439	2.162	12.4	0.6	89.6
MODEL 4	0.9297	0.455	2.159	12.7	0.6	90.1
MODEL 5	0.9344	0.456	2.113	11.3	0.6	90.1

**Table 5:** ProSA-web Z-scores of constructed 3D model and refined models.

Model	Z-score
I-TASSER model	− 3.32
Model 1	− 3.55
Model 2	− 3.32
Model 3	− 3.40
Model 4	− 3.46
Model 5	− 3.68

### Vaccine candidate disulfide engineering for stability

**Fig. 5:**
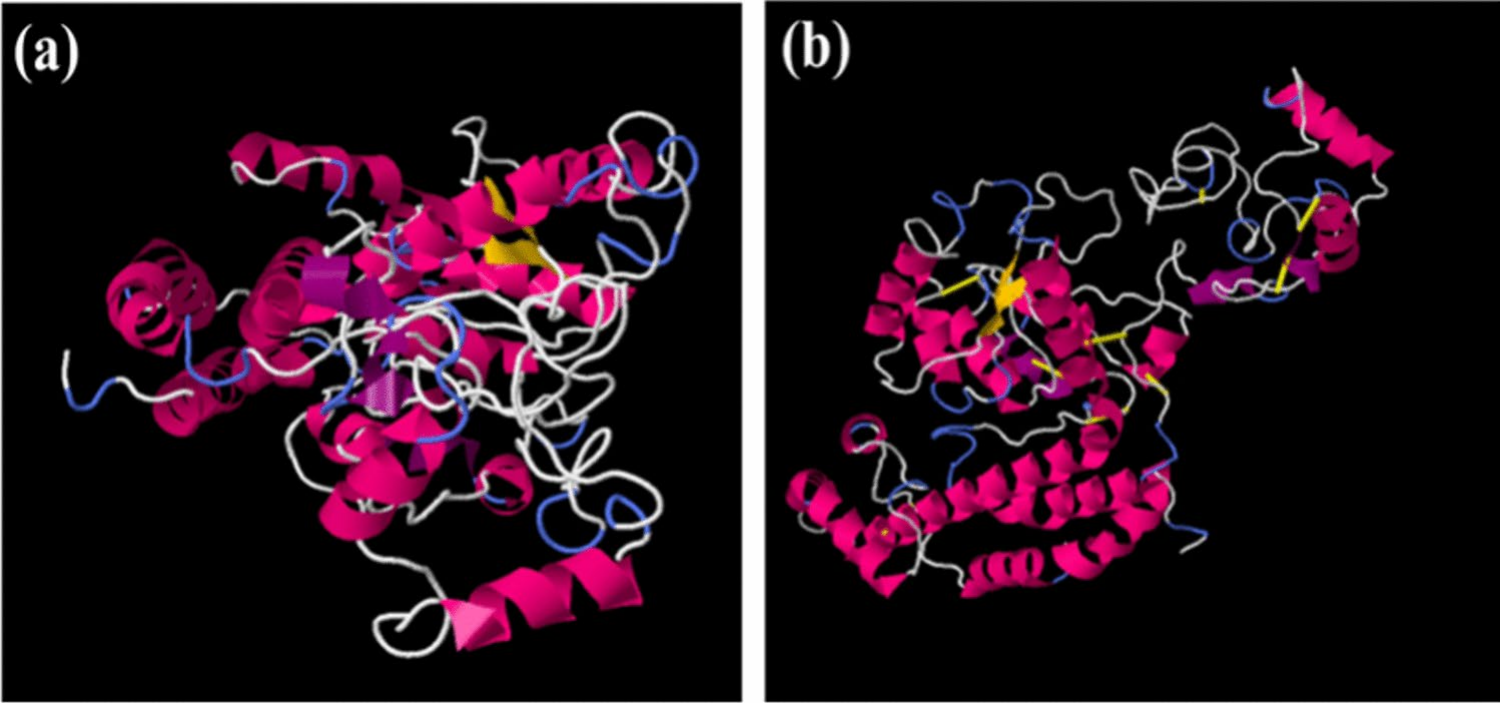
Disulfide engineering of the constructed vaccine: (a) Wild type and (b) mutant structure. The yellow colour stick displays the disulfide bonds.

A total of 35 pairs of amino acids of the vaccine were predicted to have the ability to be mutated for forming disulfide bonds, listed in **Supplementary Table S2**. Only eight residues (ALA41-ALA44, ALA194-THR324, ALA48-GLY51, VAL108-ALA113, VAL230-GLY286, ASP416-HIS425, LYS74-ALA321, LEU343-ARG346, and GLU347-THR390) with < 2.5 energy value were selected for replacement by cysteine residue (Fig. 5).

### Prediction of discontinuous B-cell epitopes

**Fig. 6:**
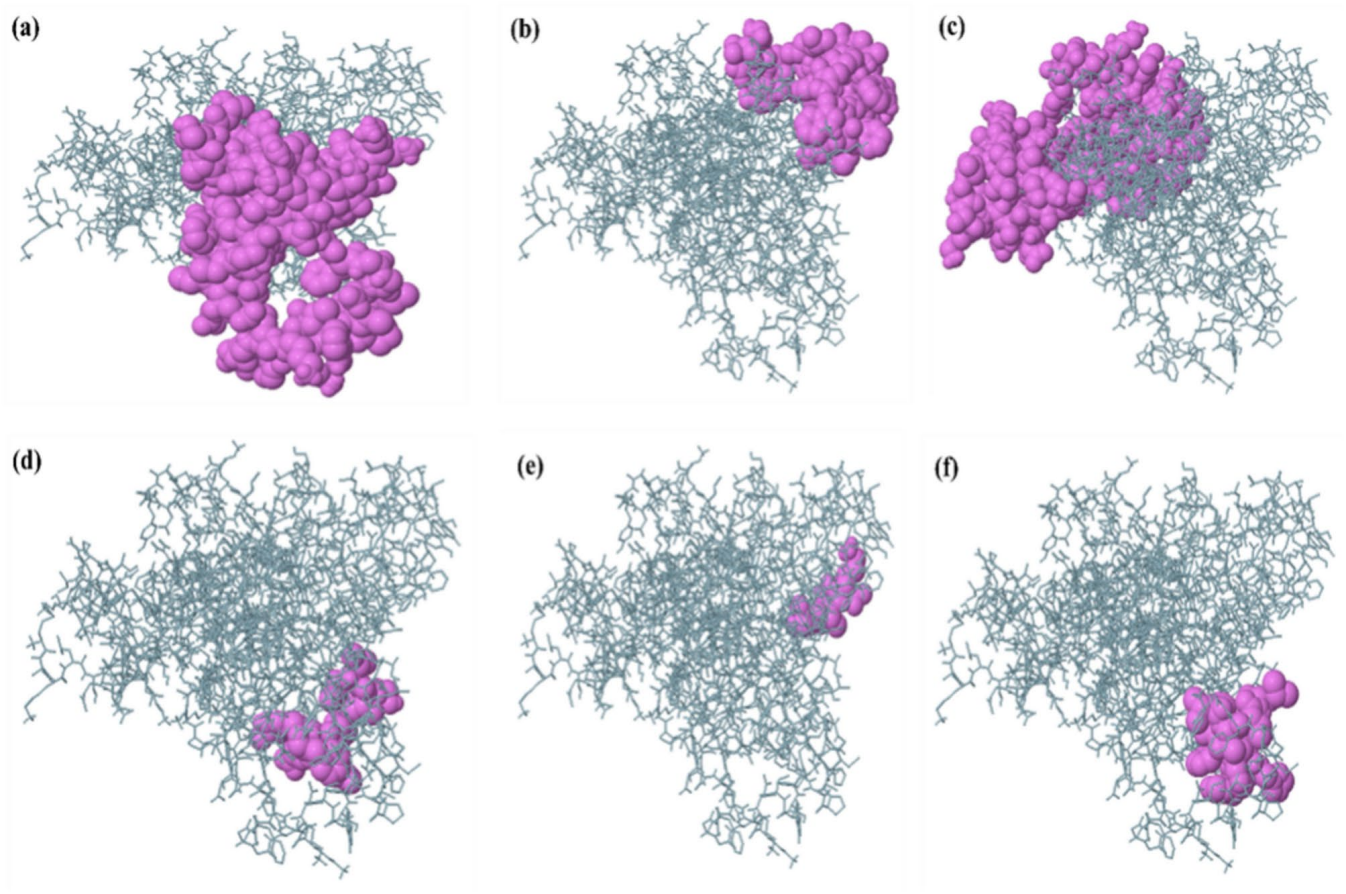
The putative B-cell epitopes present in the constructed vaccine. The violet spheres displayed epitopes containing (**a**) 70 residues (AA 335–396, AA 401–408) with a residue score of 0.802 (**b**) 36 residues (AA 133–154, AA 156–163, AA 177–182) with residue score of 0.69 (**c**) 97 residues (AA 1–5, AA 7–8, AA 11–12, AA 15–16, AA 19–20, AA 23, AA 27–28, AA 227–261, AA 270–272, AA 275–276, AA 278–296, AA 298–314, AA 316–320) with residue score of 0.683 (**d**) 14 residues (AA 52, AA 55–57, AA 59–63, AA 84–88) with residue score of 0.613 (**e**) 4 residues (AA 108–111) with residue score of 0.553 (**f**) 10 residues (AA 416–425) with residue score of 0.503.

The results from the ElliPro server demonstrated six discontinuous B-cell epitopes, as depicted in **Supplementary Table S3**. The predicted B-cell epitopes (discontinuous) showed scores that ranged from 0.503 to 0.802. The top-scored epitope comprised 70 residues, whereas the least-scored epitope comprised ten residues. The 3D structures of discontinuous B-cell epitopes present in the designed vaccine are depicted in Fig. 6.

### Prediction of cytokines-inducing potential

The IFN-epitope server was employed for predicting MHC-II binding epitopes that can induce IFN- γ. MHC-II epitopes activate T helper cells, leading to the production of interferon-gamma, which activates downstream signaling. The selected vaccine was put in the server for identifying the IFN-γ epitopes. A total of 419 potential epitopes were predicted, of which 133 showed a positive score. We found sixteen selected HTL epitopes were IL-10 inducers.

### Molecular docking investigations

**Fig. 7:**
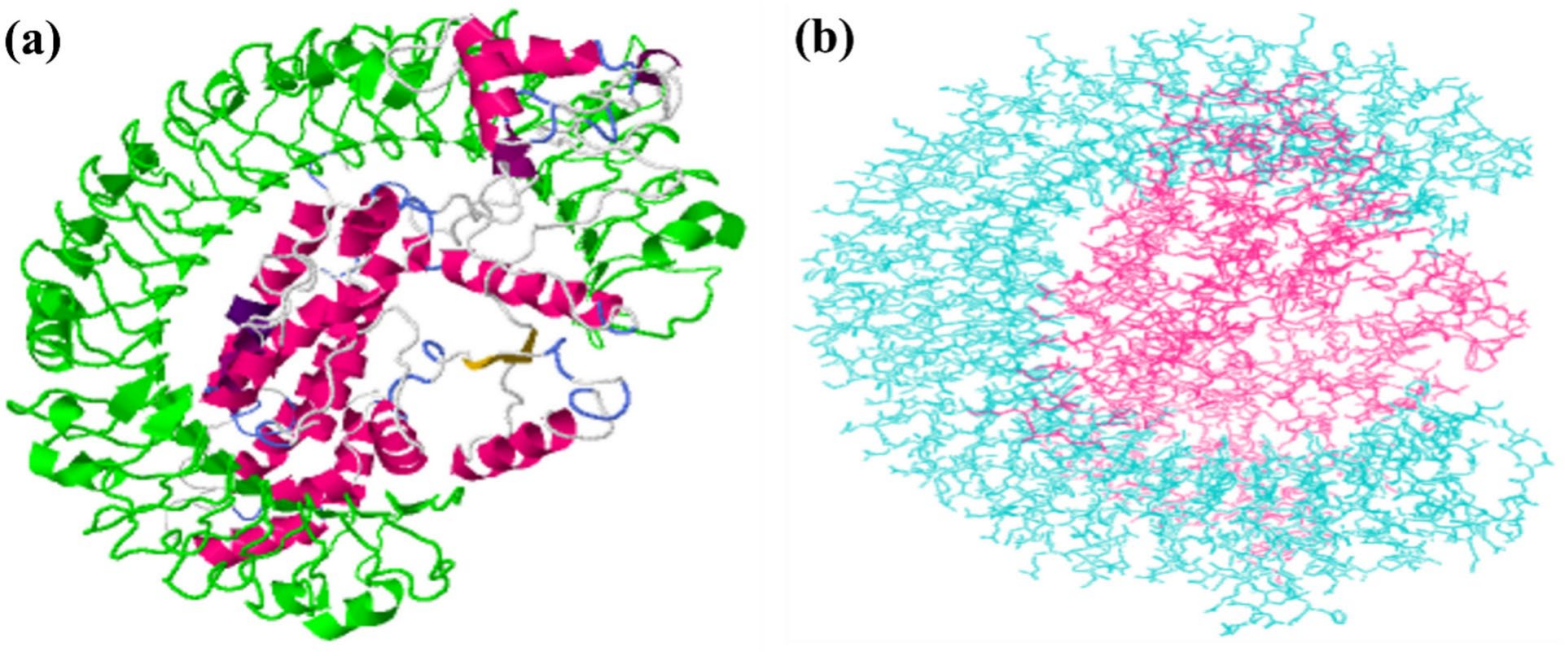
Visualization of the best-docked poses of the vaccine with TLR-3 receptor. (**a**) 3D- docking interaction of the vaccine-protein complex produced by GRAMM-X server where green coloured ribbon represents the TLR-3 receptor whereas magenta coloured ribbon shows the vaccine. (**b**) 3D- docking interaction diagram of the vaccine-protein complex visualized by maestro Schrodinger where the cyan coloured chain represents the TLR-3 receptor while the pink coloured chain shows the vaccine candidate.

The binding pockets and hydrophobic interaction sites on the final vaccine 3D model and TLR-3 receptor (PDB ID: 1ZIW) were identified using the CASTp server with a default 1.4 Å probe radius. A total of 55 binding pockets for the vaccine and 77 binding pockets for TLR-3 protein were predicted with diversified molecular surface areas and volumes. In this work, we selected a TLR-3 receptor pocket with a surface area of 2125.000 Å^2^ and volume of 13438.169 Å^3^. Similarly, for the vaccine 3D model, the binding pocket with a molecular surface area of 1214.289 Å^2^ and volume of 1410.959 Å^3^ was chosen. The GRAMMAX server performed the molecular docking of the vaccine with the TLR-3 receptor, which generated ten binding poses. Further, the docked poses were put in for calculating the docked complex’s binding affinity (ΔG). The best-docked complex of vaccine-TLR-3 protein showing the lowest binding energy (ΔG) value of −40.1 Kcal/mole at 25.0^0^ C was preferred. The Prodigy server also predicted several interfacial contacts (ICs) within a distance of 5.5 Å. The number of charged-charged, polar-polar, and apolar-apolar ICs for the vaccine was found to be 66, 28, and 68, respectively. The 3D orientation of the best docking model is visualized through the GRAMMAX server and maestro Schrodinger software (Fig. 7). The visualization of docking interactions in 2D was carried out using LIGPLOT v.4.5.3, shown in **Supplementary Figure S25**. Based on 2D interactions, hydrogen bonds (GLU8-ASN388, ASP11-ASN388, LYS14-TYR283, ALA45-ASN328, GLU58-GLU175, GLY87-THR151, ASP114-VAL34, ALA157-VAL30, ARG188-THR638, THR320-TYR462, and LYS332-GLU533) were formed between the vaccine and TLR-3 receptor. Salt bridges were observed ASP7, ASP11, GLU15, GLU68, LYS73, ASP187, and LYS332 of the final vaccine with LYS416, ARG331, LYS335, HIS410, GLU460, LYS613, and GLU533 of the TLR-3 receptor.

### Molecular dynamics simulations and binding free energy calculation

**Fig. 8:**
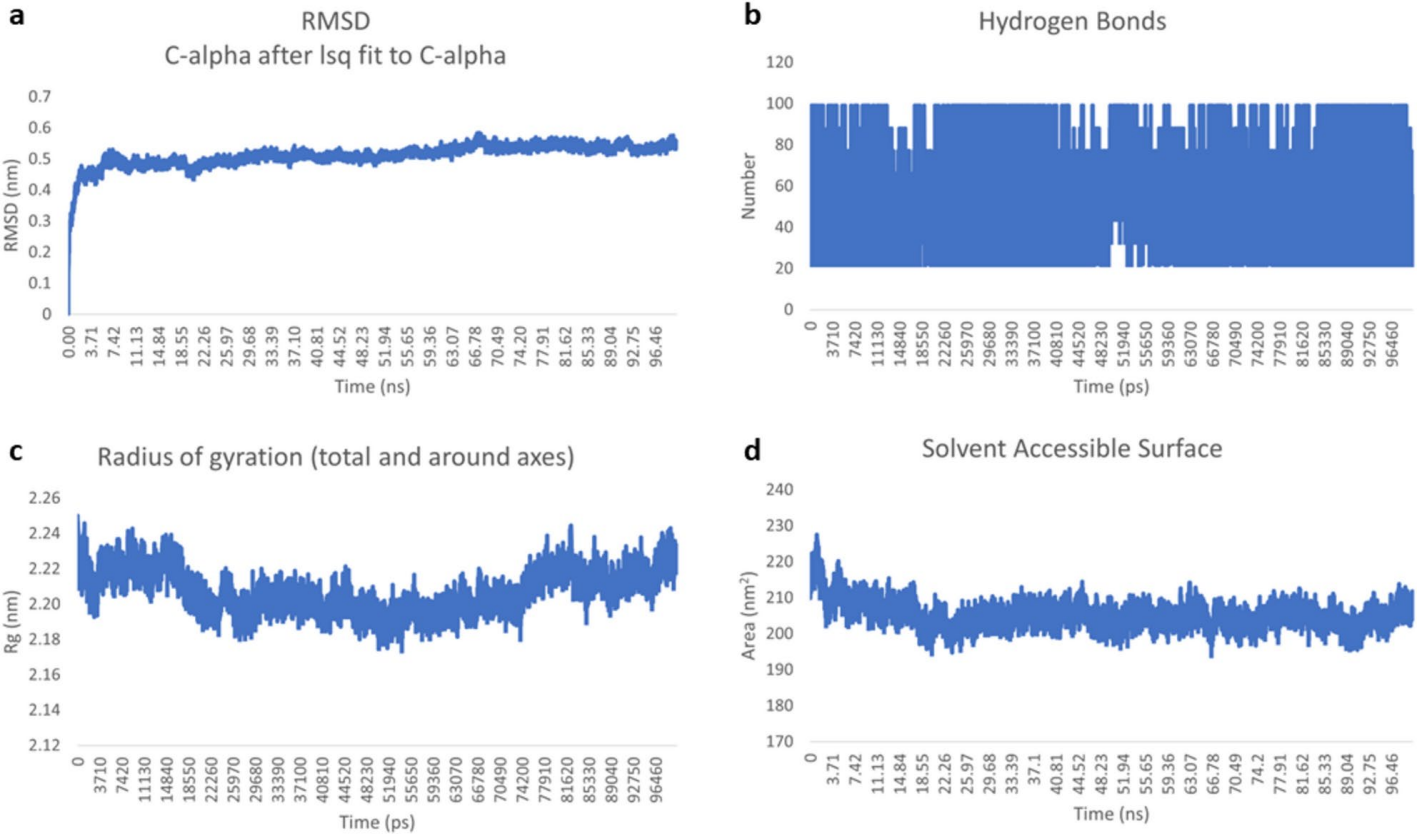
MD simulation result of vaccine and TLR3 complex. (**a**) RMSD plot of the vaccine-TLR3 complex. (**b**) Hydrogen bond plot of the vaccine-TLR3 complex. (**c**) Rg plot of vaccine-TLR3 complex. (**d**) SASA plot of vaccine-TLR3 complex.

To evaluate the stability of the designed vaccine in complex with TLR3, a 100 ns molecular dynamics (MD) simulation was performed. The initial frame served as the reference, and the root mean square deviation (RMSD) was calculated for all frames in the trajectory. RMSD quantifies the average displacement of selected atoms relative to the reference frame. The RMSD plot reveals two phases: (a) the equilibrium phase, where the ligand-protein complex reaches a stable state with no significant changes over time, and (b) the productive phase, where average deviations are analyzed. As illustrated in Fig. 8a, the vaccine-TLR3 complex reached equilibrium around 5 ns and remained stable throughout the simulation, with an average RMSD below 0.4 to 0.5 nm. This indicates that the vaccine remained within the TLR3 binding pocket. The root mean square fluctuations (RMSF) plot indicates the conformational changes that occur around the protein chain, and the value is within the range of 2.4 to 7.2 Å, as depicted in **Supplementary Figure S26. **Intermolecular hydrogen bonding is crucial for the stability of any protein-ligand complex. Figure 8b shows that most conformations of the vaccine-TLR3 complex formed 20 to 100 hydrogen bonds, which suggests that the vaccine can create and maintain binding in the active site of TLR3 protein. The radius of gyration (Rg) was calculated to assess the compactness of the complexes (Fig. 8c). Throughout the simulation, the Rg value for the vaccine-TLR3 complex varied between 2.18 to 2.24 nm. The solvent-accessible surface area (SASA) measures the protein’s exposure to solvent and helps assess changes in protein folding when bound to a ligand. Throughout the simulation, the SASA of the vaccine-TLR3 complex (Fig. 8d) ranged from 200–220 mm², further indicating the stability of the complex.

The MM-PBSA approach was employed to estimate the binding free energy of the constructed vaccine-TLR3 complex. The electrostatic energy had a significant negative value, whereas the polar solvation energy had a positive value. The contribution of each energy component is demonstrated in Table 6.

**Table 6:** Predicted free binding energies of the vaccine and TLR3 complex.

Energy	Vaccine-TLR3 complex
Van der Waals energy	− 83.240 + /− 67.747 kJ/mol
Electrostatic energy	−234.621 + /− 285.210 kJ/mol
Polar solvation energy	129.177 + /− 215.438 kJ/mol
SASA energy	−14.852 + /− 22.366 kJ/mol
Binding energy	−204.640 + /− 244.280 kJ/mol

### In silico cloning

**Fig. 9:**
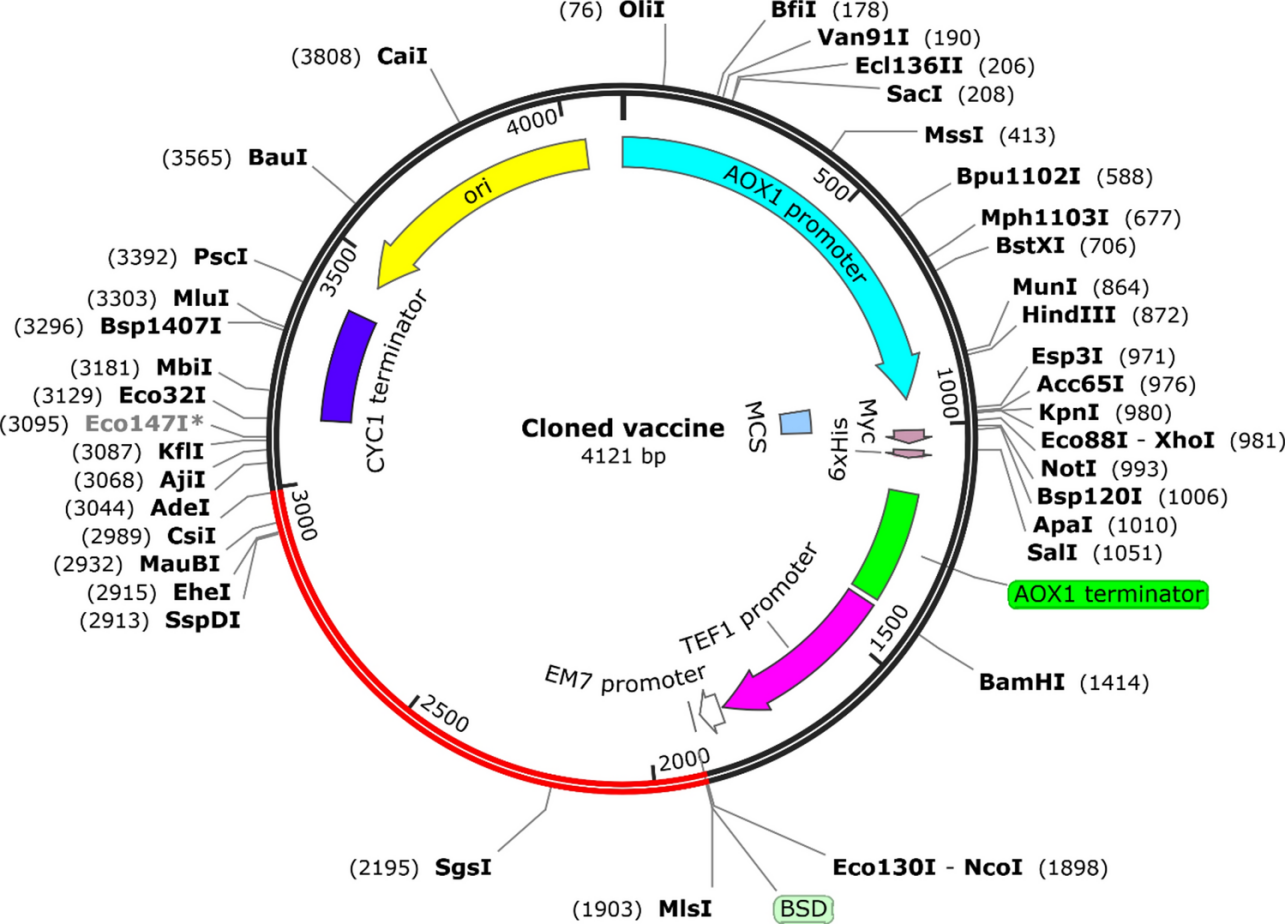
In silico restriction cloning of the final multi-epitope vaccine into the pPIC6 A expression vector. The black circle demonstrates the vector, and the red portion indicates the vaccine encoding region.

The immunoreactivity ability of a vaccine via serological assay is required to validate a developed vaccine. It is carried out by expressing the multiepitope vaccine in an appropriate host^72^. Codon bias arises when two or more codons encode the same amino acid in different animals. As a consequence, codon adaptation was employed in this study to estimate the optimal codon of the vaccine sequence for encoding in a particular organism^73^. Codon adaptation was performed using the VectorBuider server by selecting *E.coli* str. K-12 substrate. MG1655 as a target expression system. The fundamental properties, such as CAI value and GC content of the modified sequence, were 0.93 and 57.01%, respectively. Finally, the optimized codon sequences of the constructed vaccine with two recognition enzyme sites (MlsI and AdeI) were inserted into the pPIC6 A vector using the SnapGene tool. A successful clone of 4121bp was obtained (Fig. 9).

### Immune simulation analysis

#### Immune cell populations

**Fig. 10:**
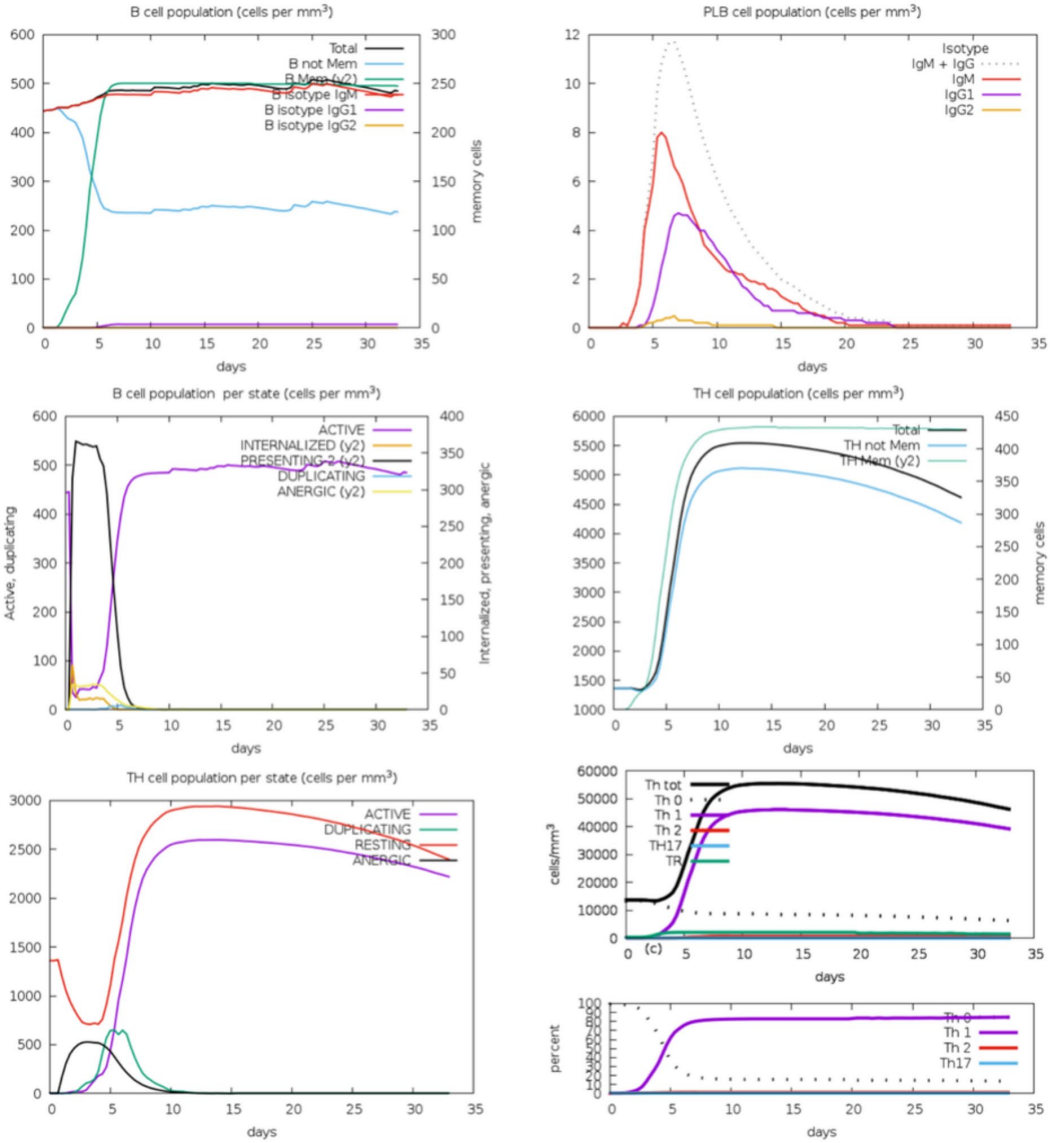
Immune cell counts shown. Legend: Act = active, Intern = internalized the Ag, Pres II = presenting on MHC II, Dup = in the mitotic cycle, Anergic = anergic, Resting = not active.

The immune simulation indicated robust activation and proliferation of various immune cell populations. The counts of active cells, internalized antigen-presenting cells (APCs), and cells presenting antigens on MHC class II molecules were notably high. The result suggests efficient antigen uptake and presentation, essential for initiating and sustaining adaptive immune responses. Specifically, active B cells and cytotoxic T cells (CTLs) showed significant increases, indicating a potent cellular and humoral immune response to the multi-epitope vaccine (Fig. 10)

#### Antigen presentation and memory formation

Figures from the simulation demonstrated a substantial number of APCs presenting the antigen on MHC II molecules (**Supplementary Figure S27**). This is crucial for activating CD4+ helper T cells, which are necessary for B cell activation and differentiation into plasma and memory B cells. The high number of mitotic cells indicates ongoing proliferation, which is vital for generating a large pool of effector and memory cells.

#### Antibody production

The simulation results highlighted significant production of various immunoglobulin (Ig) isotypes, with IgM being the first to appear, followed by IgG (**Supplementary Figure S28**). The presence of these antibodies in large quantities suggests that the vaccine effectively stimulated a solid humoral response. The formation of immunocomplexes further indicates that the antibodies produced were functional and capable of binding to the antigen.

#### Cytokine and interleukin response

The concentration of cytokines and interleukins was monitored, particularly concerning IL-2, a crucial growth factor for T cells (**Supplementary Figure S29**). The results showed an elevated IL-2 level, indicating a robust proliferative response. Other cytokines, such as IFN-γ and IL-4, were also elevated, suggesting a balanced Th1/Th2 response, beneficial for both cellular and humoral immunity.

## Discussion

The present study is very novel and is the first of its kind where different serovars of *C. trachomatis* have been used to design a broad-spectrum multi-epitope vaccine which might confer immunity against different *C. trachomatis* strains like A, B, D, E, L1, and L2. The immune simulation results underscore the potential efficacy of the designed multi-epitope vaccine targeting *C. trachomatis* serovars. The robust activation of both humoral and cellular immune responses is a promising indicator of the vaccine’s effectiveness. The significant proliferation of cytotoxic T cells (CTLs) and B cells, along with the high number of active antigen-presenting cells (APCs), demonstrates comprehensive immune activation. This is crucial for developing immunity, as both arms of the adaptive immune system are engaged, providing a broad defense mechanism against the pathogen.

The interaction between the vaccine and TLR-3 receptor, characterized by molecular docking and dynamics simulations, further supports the vaccine’s potential. The stable binding affinity and the formation of multiple hydrogen bonds and salt bridges suggest a strong and stable interaction, essential for effective immune activation. This aligns with previous studies highlighting the importance of stable antigen-TLR interactions for robust immune responses^74^. Efficient antigen presentation by APCs, as evidenced by the simulation results, is critical for the activation of CD4+ helper T cells. This activation is necessary for B cell differentiation into plasma cells and memory B cells, ensuring both immediate and long-term immune protection. The high proliferation rate of these cells indicates the vaccine’s potential to generate a substantial pool of effector and memory cells, crucial for lasting immunity. This aspect of immune response is well-documented, emphasizing the role of helper T cells in orchestrating the adaptive immune response^75^.

The significant production of various immunoglobulin (Ig) isotypes, particularly IgM and IgG, highlights the vaccine’s ability to induce a strong humoral response. IgM, appearing first, indicates an initial response, while IgG, appearing subsequently, suggests long-term humoral immunity. The formation of immunocomplexes suggests functional antibodies capable of neutralizing the antigen, which is a critical aspect of vaccine efficacy. These findings are consistent with the role of antibodies in pathogen neutralization and clearance^76^. The elevated levels of IL-2, IFN-γ, and IL-4 indicate a balanced Th1/Th2 response, which is beneficial for a comprehensive immune defense. IL-2 is crucial for T cell proliferation, while IFN-γ and IL-4 are essential for activating cellular and humoral immunity, respectively. A balanced Th1/Th2 response ensures that intracellular and extracellular pathogens are effectively targeted, providing a well-rounded immune defense. This balanced cytokine response is supported by literature as essential for effective vaccination^77^.

Targeting multiple epitopes across different serovars of *C. trachomatis*enhances the vaccine’s potential for broad protection. The conservation analysis indicating a 65.41% conservancy across serovars suggests that the vaccine can provide immunity against a wide range of strains. This broad-spectrum efficacy is particularly important for pathogens with high genetic variability, ensuring the vaccine’s relevance across different populations and geographic regions. The importance of epitope diversity in vaccine design is well-established, supporting the strategy employed in this study^78^. The physicochemical characterization and stability analyses indicate that the vaccine construct is stable and likely to maintain its structure and function in various conditions. The antigenicity and non-allergenicity predictions further suggest that the vaccine is safe and capable of eliciting a strong immune response without causing adverse reactions. These characteristics are essential for a viable vaccine candidate and align with the criteria for successful vaccine development^79^. The PC parameters are in line with previous published literature^80,81^.

Comparing these findings with other published research, it is evident that the integration of computational tools in vaccine design is becoming increasingly significant. For instance, studies on multi-epitope vaccines against SARS-CoV-2 have demonstrated similar approaches in utilizing antigenic, non-toxic, and non-allergenic B-cell and T-cell epitopes from conserved regions of viral proteins^72,82^. These studies have shown that stable and strong interactions with toll-like receptors (TLRs) are crucial for effective immune activation^83^. Additionally, the importance of robust computational methods in predicting immune responses accurately has been highlighted in various bioinformatic studies^84^. Despite our best efforts to select the best epitopes, this study is limited by being computational in nature. The algorithms used to predict epitopes, other PC properties of the vaccine, and 2D and 3D structures are still developing and, hence, need experimental validation through *in vitro* studies. and certain limitations remain that future research should address.

## Conclusion

This study uses major outer membrane protein sequences to present an in silico muti-epitope vaccine design against C. trachomatis. The novelty of this study lies in using different serovars of *C. trachomatis* to design a broad-spectrum multi-epitope vaccine which might confer immunity against different *C. trachomatis* strains like A, B, D, E, L1, and L2. The vaccine construct is characterized by favourable antigenic, non-allergic, and highly soluble in water without toxic behaviour. The designed vaccine exhibits strong interaction with the TLR3 receptor and is dynamically stable. Further, the immune simulation study of the designed multi-epitope vaccine against *C. trachomatis* demonstrates its potential effectiveness in inducing a robust and balanced immune response. The vaccine’s ability to stimulate both cellular and humoral immunity, coupled with the formation of memory cells, suggests that it could provide long-lasting protection against multiple serovars of the pathogen. These findings support further vaccine development and in vivo testing to validate its efficacy and safety.

## Supplementary Information

Supplementary Information.

## Data Availability

The datasets used in the manuscript are publicly available from the repositories below: (1) Repository Name: UniProt [ Link to the repository: https://www.uniprot.org/; (2) Macromolecular structure of TLR3 [link to the repository: https://www.rcsb.org/structure/1ZIW] and deposited initially from the article, https://doi.org/10.1126/science.1115253 and (3) other datasets generated during the current study can be made available from the corresponding author upon reasonable request.
