# Supplementary material for: Immunoinformatics design of a multi-epitope vaccine for Chlamydia trachomatis major outer membrane proteins
Source: Sci Rep. 2024 Dec 2;14:29919. doi: 10.1038/s41598-024-81736-w (PMC11612408; doi:10.1038/s41598-024-81736-w)
Supplement: Supplementary file 1 — Supplementary Information. [file 41598_2024_81736_MOESM1_ESM.docx]

Supplementary Material

**Immunoinformatics Design of a Multi-Epitope Vaccine for *Chlamydia trachomatis* Outer Membrane Proteins**

Seema Shetty^1,2^, Swagatika Dash^3^, Avinash Kumar^4^, Shashidhar Vishwanath^1^, Suvarna G. Kini,^3,5*^, Angela Brand^2,6,7^

^1^Department of Microbiology, Kasturba Medical College, Manipal, Manipal Academy of Higher Education, Madhav Nagar, Manipal 576104, Karnataka, India. ^2^Faculty of Health, Medicine and Life Sciences, Maastricht University, Maastricht 6229, GT, The Netherlands. ^3^Department of Pharmaceutical Chemistry, Manipal College of Pharmaceutical Sciences, Manipal Academy of Higher Education, Manipal, Karnataka, India 576104. ^4^Department of Medical Affairs, Curie Sciences Private Limited, Samastipur, Bihar, India 848125. ^5^Manipal Mc Gill Centre for Infectious Diseases, Prasanna School of Public Health, Manipal Academy of Higher Education, Manipal, Karnataka, India 576104. ^6^Prasanna School of Public Health, Manipal Academy of Higher Education, Manipal, India. ^7^United Nations University – Maastricht Economics and Social Research Institute on Innovation and Technology (UNU-MERIT), Maastricht 6211, AX, The Netherlands.

***Corresponding author:** Dr. Suvarna. G Kini, Department of Pharmaceutical Chemistry, Manipal College of Pharmaceutical Sciences, Manipal Academy of Higher Education, Manipal, Karnataka, India 576104. Email: [suvarna.gk@manipal.edu](mailto:suvarna.gk@manipal.edu)

**Contents**

**Supplementary Table S1:** List of selected epitopes from MOMP of different serovars with their antigenicity, allergenicity, and toxicity features.

**Supplementary Table S2:** Disulfide engineering of the final multiepitope vaccine candidate.

**Supplementary Table S3:** Discontinuous B-cell epitopes with their scores predicted by ElliPro.

**Supplementary Figure S1:** 3D interaction diagram of HEWQASLAL with HLA-A*02:01 (PDB ID 4U6Y).

**Supplementary Figure S2:** 3D interaction diagram of HEWQASLAL with HLA-A*24:02 (PDB ID 8SBK).

**Supplementary Figure S3:** 3D interaction diagram of HEWQASLAL with HLA A*02:06 (PDB ID 3OXR).

**Supplementary Figure S4:** 3D interaction diagram of HEWQASLAL with HLA-B*40:02 (PDB ID 5IEH).

**Supplementary Figure S5:** 3D interaction diagram of SLDQSVVEL with HLA-A*02:01 (PDB ID 4U6Y).

**Supplementary Figure S6:** 3D interaction diagram of SLDQSVVEL with HLA-A*24:02 (PDB ID 8SBK).

**Supplementary Figure S7:** 3D interaction diagram of SLDQSVVEL with HLA A*02:06 (PDB ID 3OXR).

**Supplementary Figure S8:** 3D interaction diagram of SLDQSVVEL with HLA-B*40:02 (PDB ID 5IEH).

**Supplementary Figure S9:** 3D interaction diagram of AESVPNMSF with HLA-A*02:01 (PDB ID 4U6Y).

**Supplementary Figure S10:** 3D interaction diagram of AESVPNMSF with HLA-A*24:02 (PDB ID 8SBK).

**Supplementary Figure S11:** 3D interaction diagram of AESVPNMSF with HLA A*02:06 (PDB ID 3OXR).

**Supplementary Figure S12:** 3D interaction diagram of AESVPNMSF with HLA-B*40:02 (PDB ID 5IEH).

**Supplementary Figure S13:** 3D interaction diagram of LYTDTAFSW with HLA-A*02:01 (PDB ID 4U6Y).

**Supplementary Figure S14:** 3D interaction diagram of LYTDTAFSW with HLA-A*24:02 (PDB ID 8SBK).

**Supplementary Figure S15:** 3D interaction diagram of LYTDTAFSW with HLA A*02:06 (PDB ID 3OXR).

**Supplementary Figure S16:** 3D interaction diagram of LYTDTAFSW with HLA-B*40:02 (PDB ID 5IEH).

**Supplementary Figure S17:** 3D interaction diagram of FVFDRVLQT with HLA-A*02:01 (PDB ID 4U6Y).

**Supplementary Figure S18:** 3D interaction diagram of FVFDRVLQT with HLA-A*24:02 (PDB ID 8SBK).

**Supplementary Figure S19:** 3D interaction diagram of FVFDRVLQT with HLA A*02:06 (PDB ID 3OXR).

**Supplementary Figure S20:** 3D interaction diagram of FVFDRVLQT with HLA-B*40:02 (PDB ID 5IEH).

**Supplementary Figure S21:** 3D interaction diagram of LYTDTTFAW with HLA-A*02:01 (PDB ID 4U6Y).

**Supplementary Figure S22:** 3D interaction diagram of LYTDTTFAW with HLA-A*24:02 (PDB ID 8SBK).

**Supplementary Figure S23:** 3D interaction diagram of LYTDTTFAW with HLA A*02:06 (PDB ID 3OXR).

**Supplementary Figure S24:** 3D interaction diagram of LYTDTTFAW with HLA-B*40:02 (PDB ID 5IEH).

**Supplementary Figure S25:** 2D-interaction diagram (Zoom form) of the protein-protein complex generated by the GRAMM-X server where chain A is from the TLR-3 receptor and chain C is the vaccine construct.

**Supplementary Figure S26:** RMSF plot of the vaccine-TLR3 complex.

**Supplementary Figure S27:** Immune cells count: DC, dendritic cells; EP, eosinophil; NK, natural killer cells; TC, cytotoxic T-cells.

**Supplementary Figure S28:** The virus, the immunoglobulins, and the immunocomplexes.

**Supplementary Figure S29:** Concentration of cytokines and interleukins. Inset plot shows danger signal together with leukocyte growth factor IL-2.

**Supplementary Table S1:** List of selected epitopes from MOMP of different serovars with their antigenicity, allergenicity, and toxicity features.

| **Serovars name** | **Epitope type** | **Epitope name** | **Antigenicity** | **Allergenicity** | **Toxicity** |
| --- | --- | --- | --- | --- | --- |
| A | CTL | HEWQASLAL | 0.7912 (antigen) | Non-allergen | Non-toxin |
|  | HTL | ETRLIDERAAHVNAQ | 1.1841 (antigen) | Non-allergen | Non-toxin |
|  | B-cell | TGNATAPTTLTARENP | 0.6681 (antigen) | Non-allergen | Non-toxin |
| B | CTL | SLDQSVVEL | 0.6977  (antigen) | Non-allergen | Non-toxin |
|  | HTL | TMQIVSLQLNKMKSR | 0.8161 (antigen) | Non-allergen | Non-toxin |
|  | B-cell | TGNAVAPSTLTARENP | 0.6379 (antigen) | Allergen | Non-toxin |
| D | CTL | AESVPNMSF | 0.7916 (antigen) | Non-allergen | Non-toxin |
|  | HTL | DTMQIVSLQLNKMKS | 0.4241 (non-antigen) | Non-allergen | Non-toxin |
|  | B-cell | EGFGGDPCDPCATWCD | 0.0705 (antigen) | Non-allergen | Non-toxin |
| E | CTL | LYTDTAFSW | 0.5897 (antigen) | Non-allergen | Non-toxin |
|  | HTL | ADTIRIAQPKSATAI | 0.6423 (antigen) | Non-allergen | Non-toxin |
|  | B-cell | EGFGGDPCDPCTTWCD | 0.8546  (antigen) | Non-allergen | Non-toxin |
| L1 | CTL | FVFDRVLQT | 0.7382 (antigen) | Non-allergen | Non-toxin |
|  | HTL | NKEFQMGAKPTATTG | 1.0498 (antigen) | Non-allergen | Non-toxin |
|  | B-cell | TGTKDASIDYHEWQAS | 1.1225 (antigen) | Non-allergen | Non-toxin |
| L2 | CTL | LYTDTTFAW | 0.9172 (antigen) | Allergen | Non-toxin |
|  | HTL | ADTIRIAQPKSATTV | 0.7533 (antigen) | Non-allergen | Non-toxin |
|  | B-cell | AQPKSATTVFDVTTLN | 0.5793 (antigen) | Non-allergen | Non-toxin |


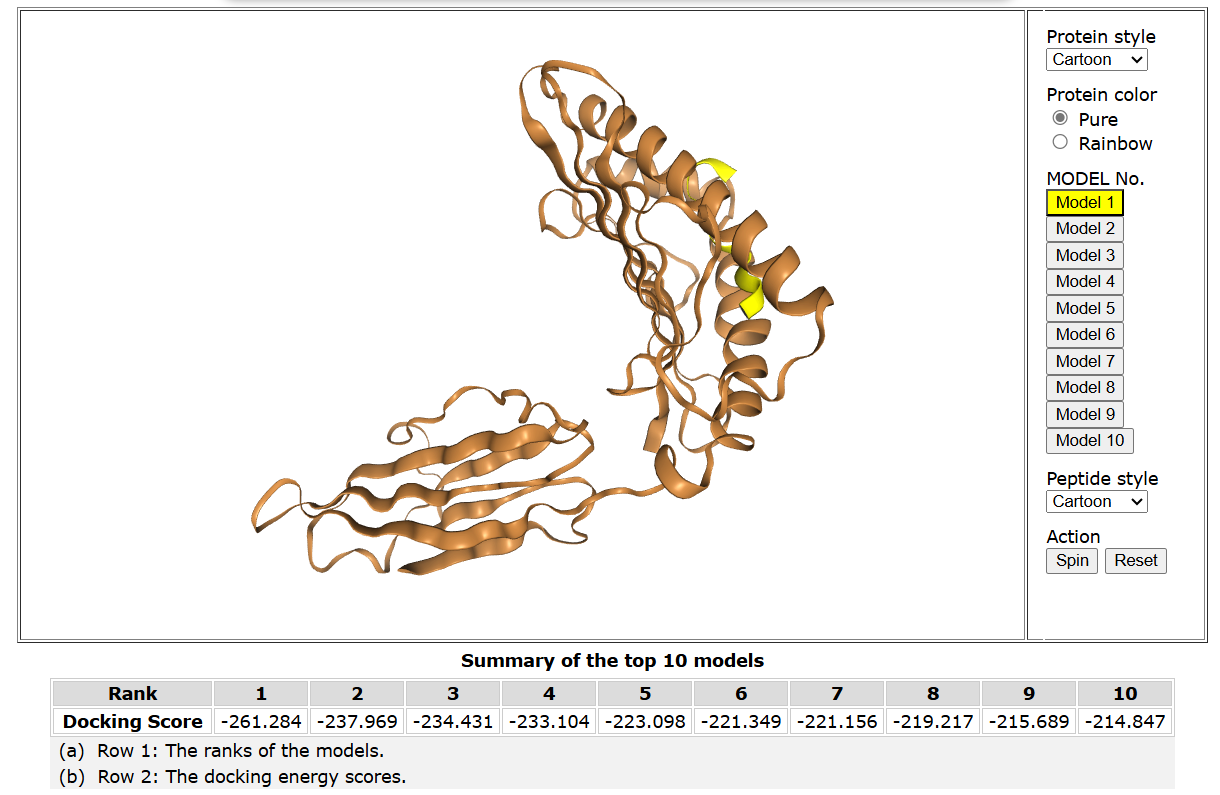


**Supplementary Figure S1:** 3D interaction diagram of HEWQASLAL with HLA-A*02:01 (PDB ID 4U6Y).


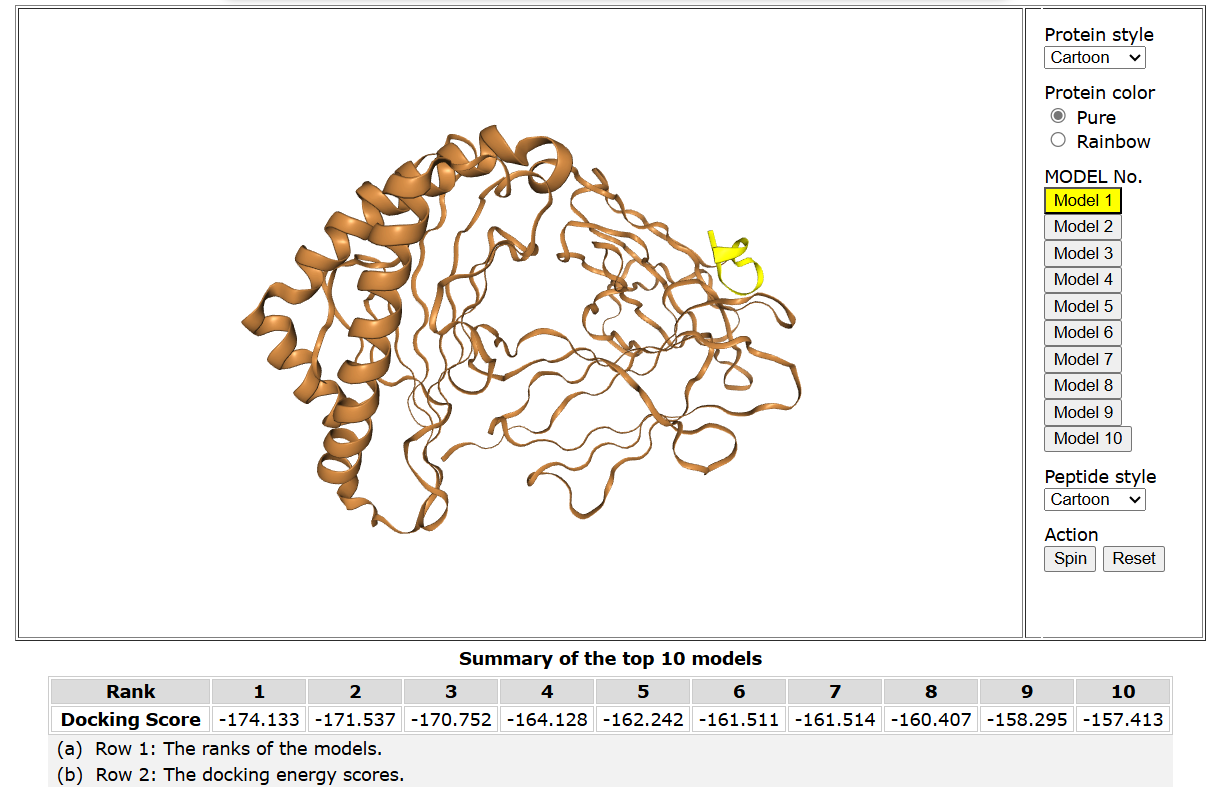


**Supplementary Figure S2:** 3D interaction diagram of HEWQASLAL with HLA-A*24:02 (PDB ID 8SBK).


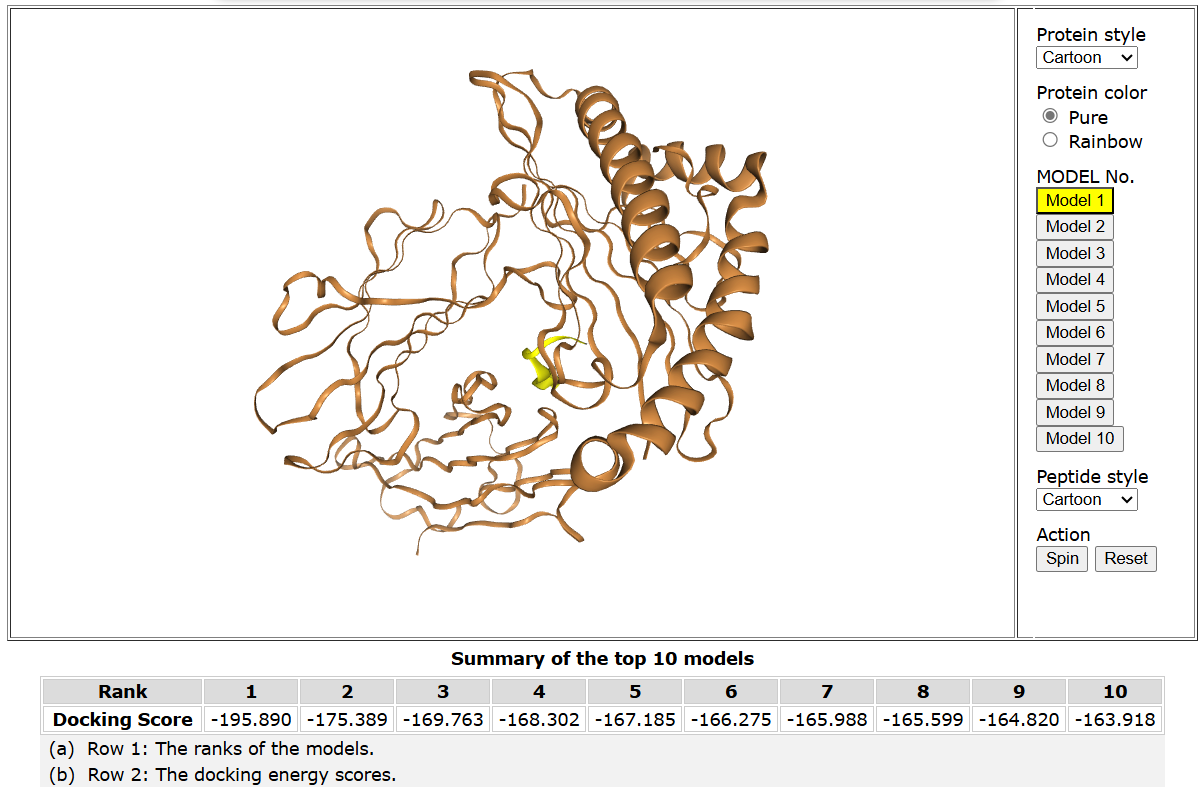


**Supplementary Figure S3:** 3D interaction diagram of HEWQASLAL with HLA A*02:06 (PDB ID 3OXR).


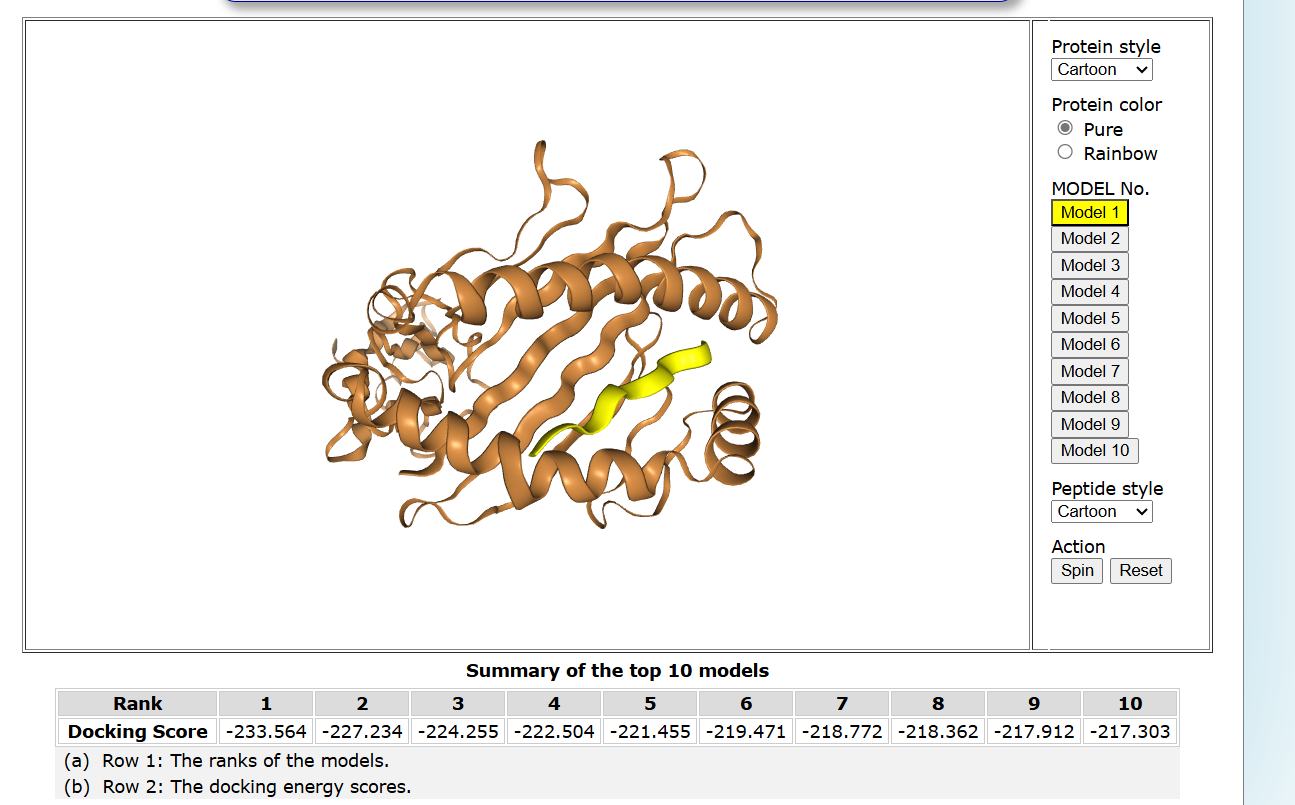


**Supplementary Figure S4:** 3D interaction diagram of HEWQASLAL with HLA-B*40:02 (PDB ID 5IEH).


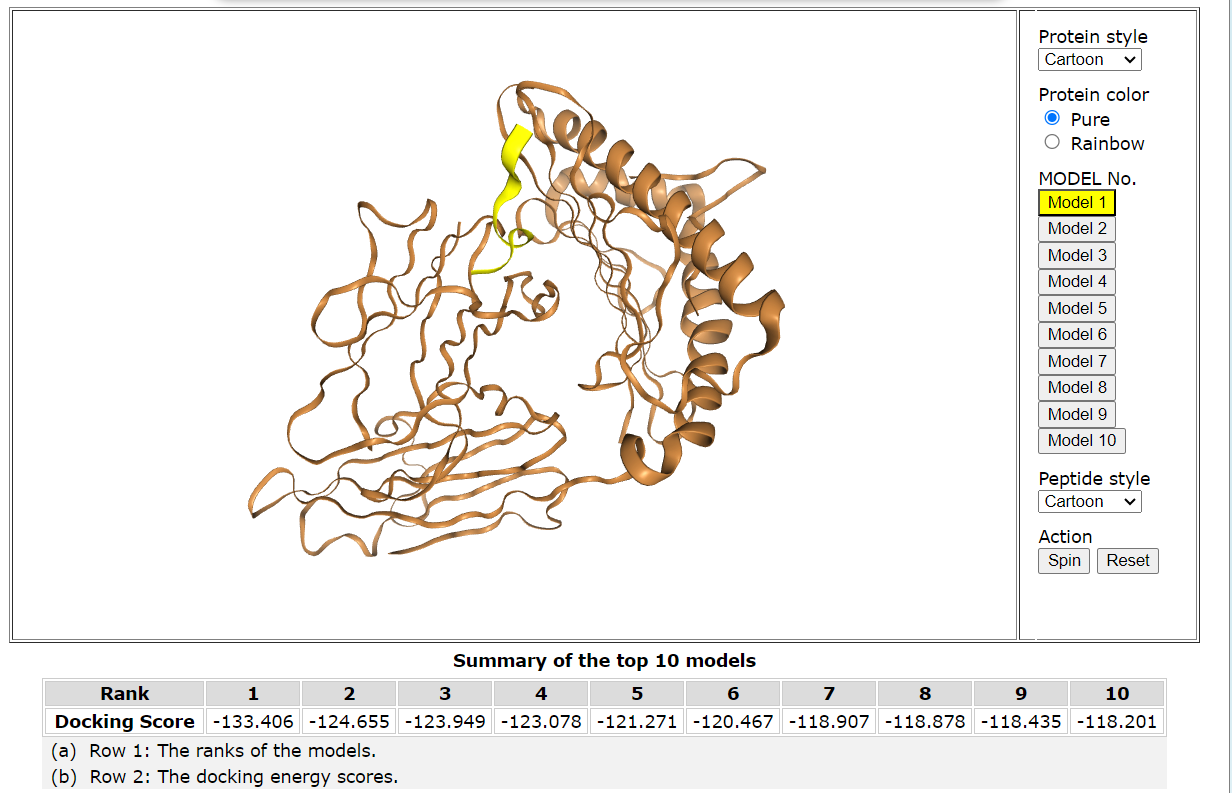


**Supplementary Figure S5:** 3D interaction diagram of SLDQSVVEL with HLA-A*02:01 (PDB ID 4U6Y).


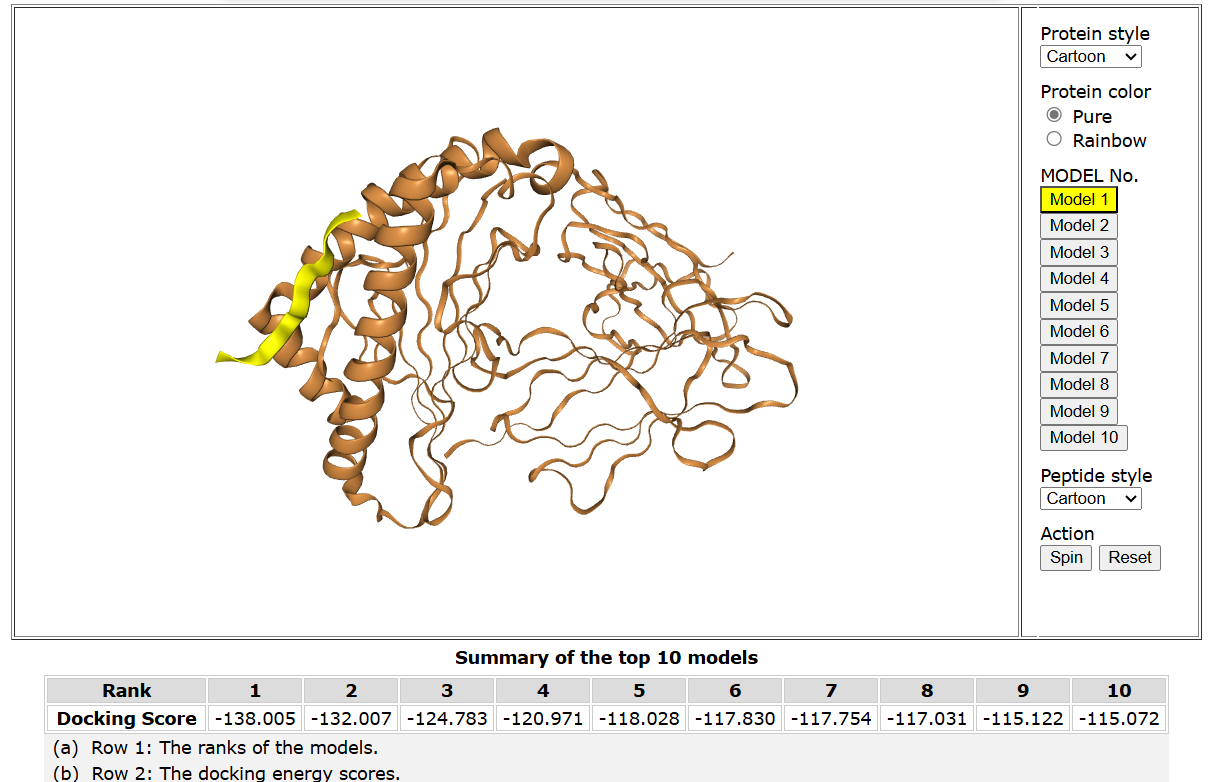


**Supplementary Figure S6:** 3D interaction diagram of SLDQSVVEL with HLA-A*24:02 (PDB ID 8SBK).


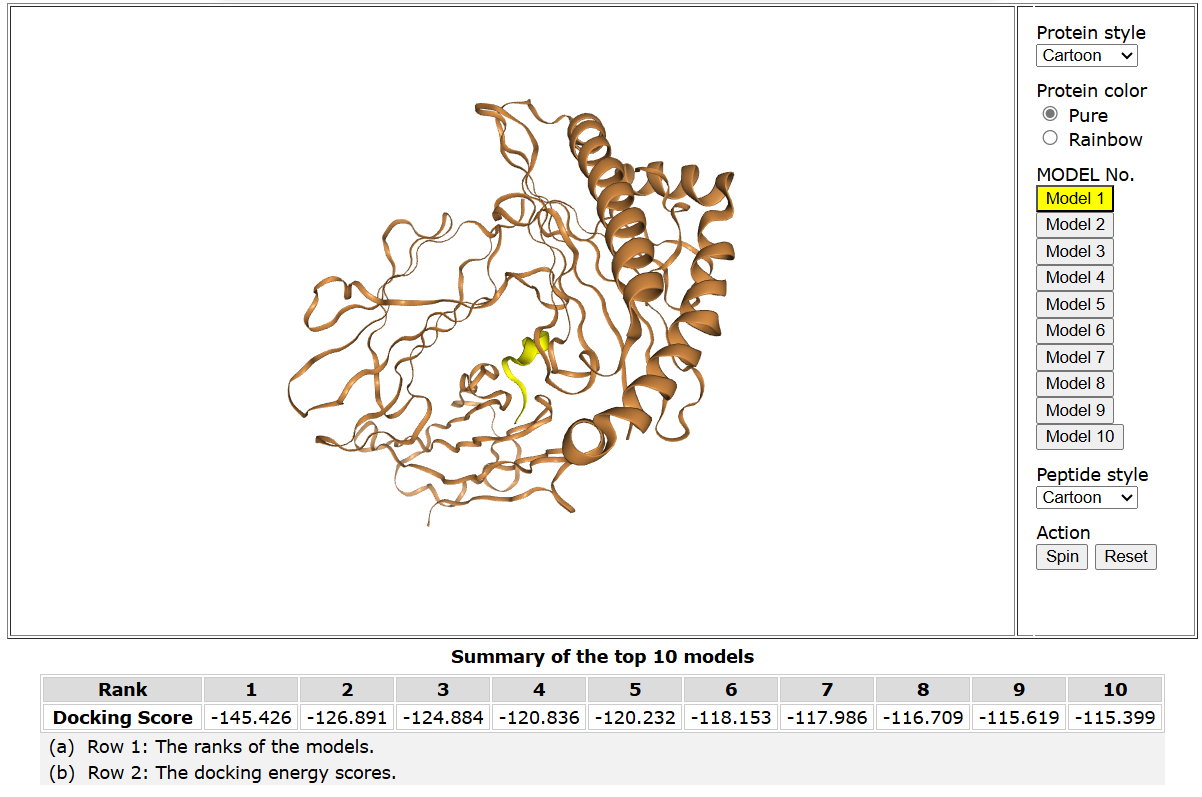


**Supplementary Figure S7:** 3D interaction diagram of SLDQSVVEL with HLA A*02:06 (PDB ID 3OXR).


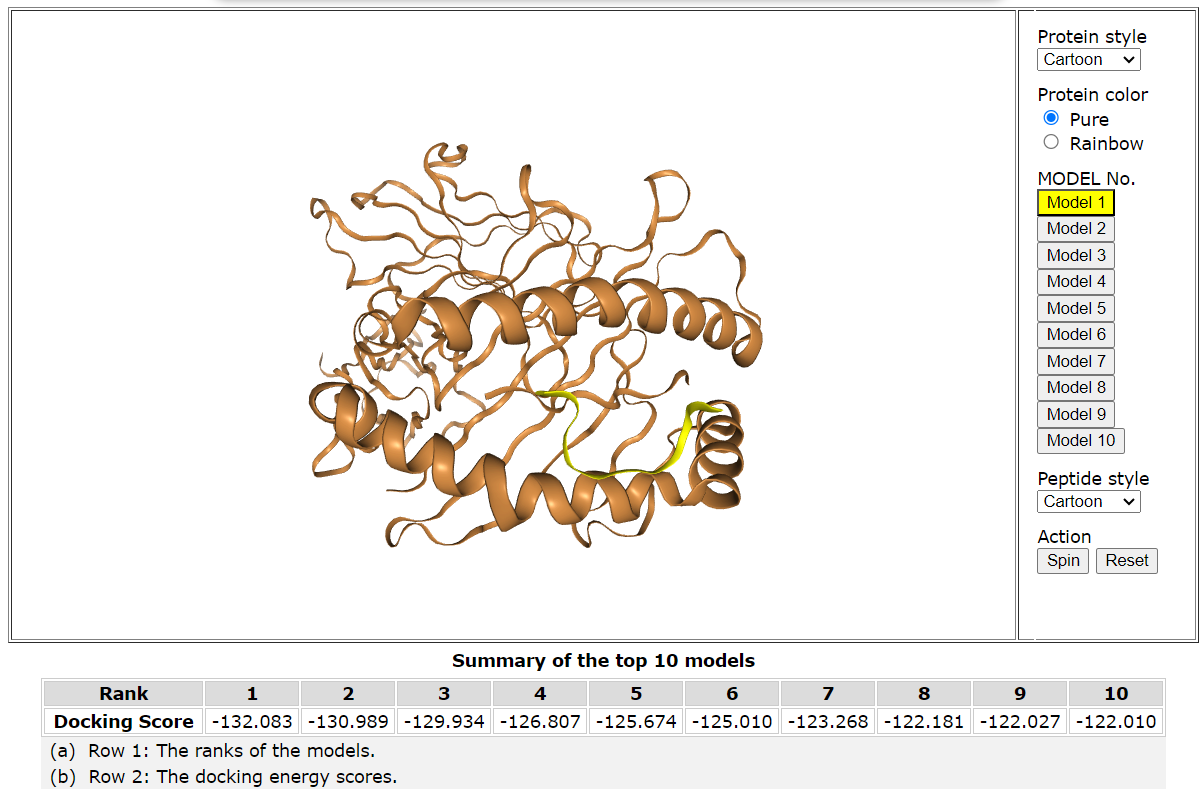


**Supplementary Figure S8:** 3D interaction diagram of SLDQSVVEL with HLA-B*40:02 (PDB ID 5IEH).


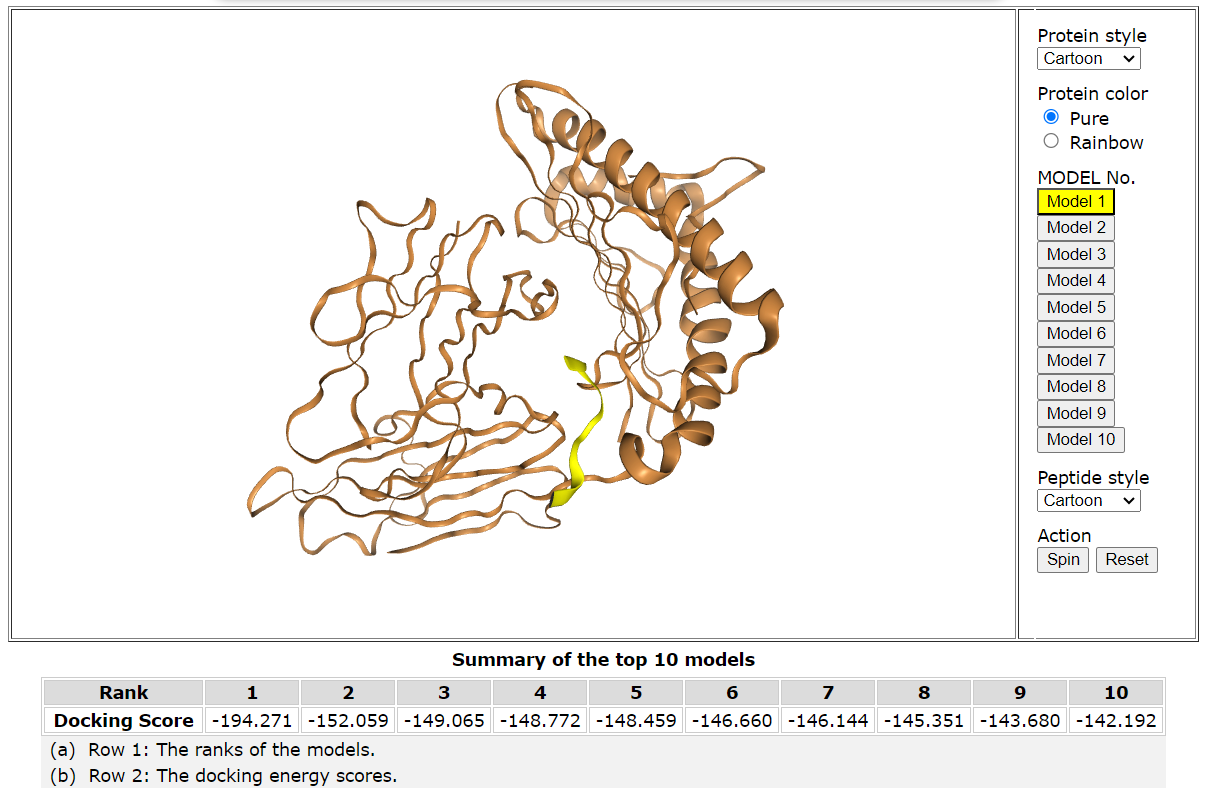


**Supplementary Figure S9:** 3D interaction diagram of AESVPNMSF with HLA-A*02:01 (PDB ID 4U6Y).


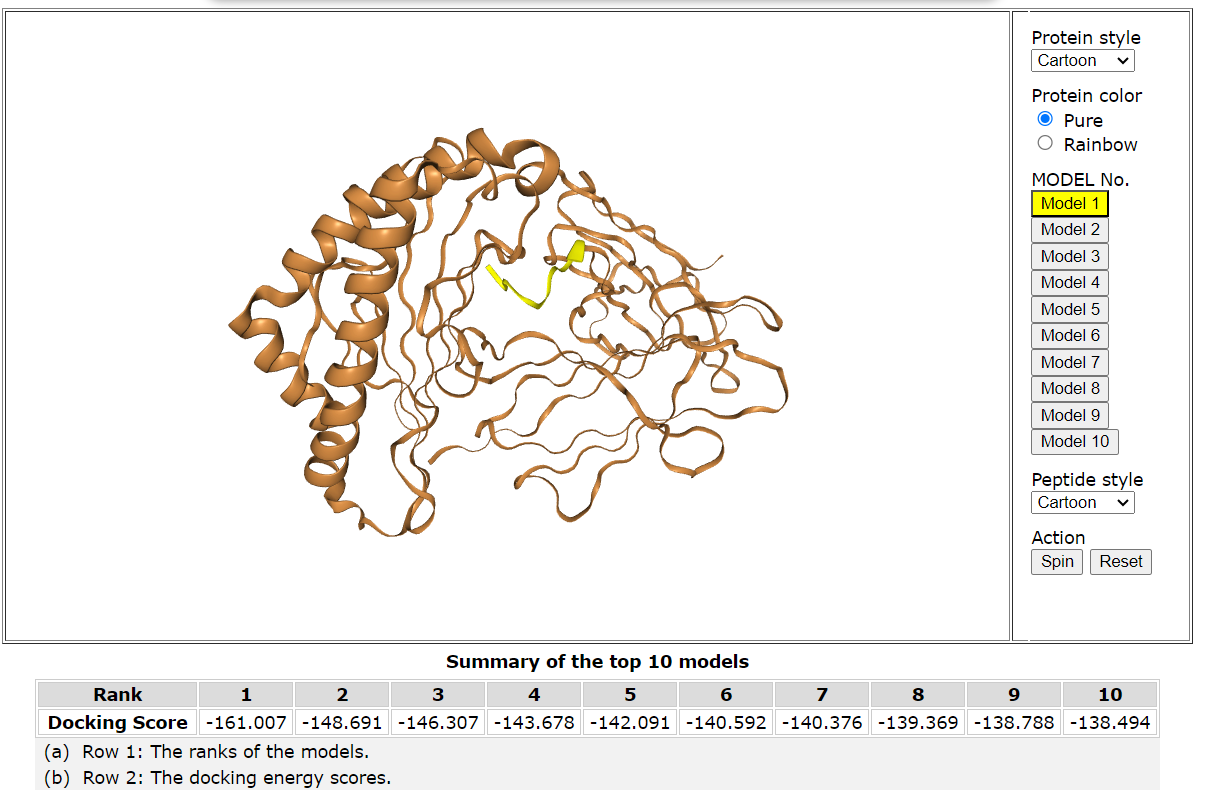


**Supplementary Figure S10:** 3D interaction diagram of AESVPNMSF with HLA-A*24:02 (PDB ID 8SBK).


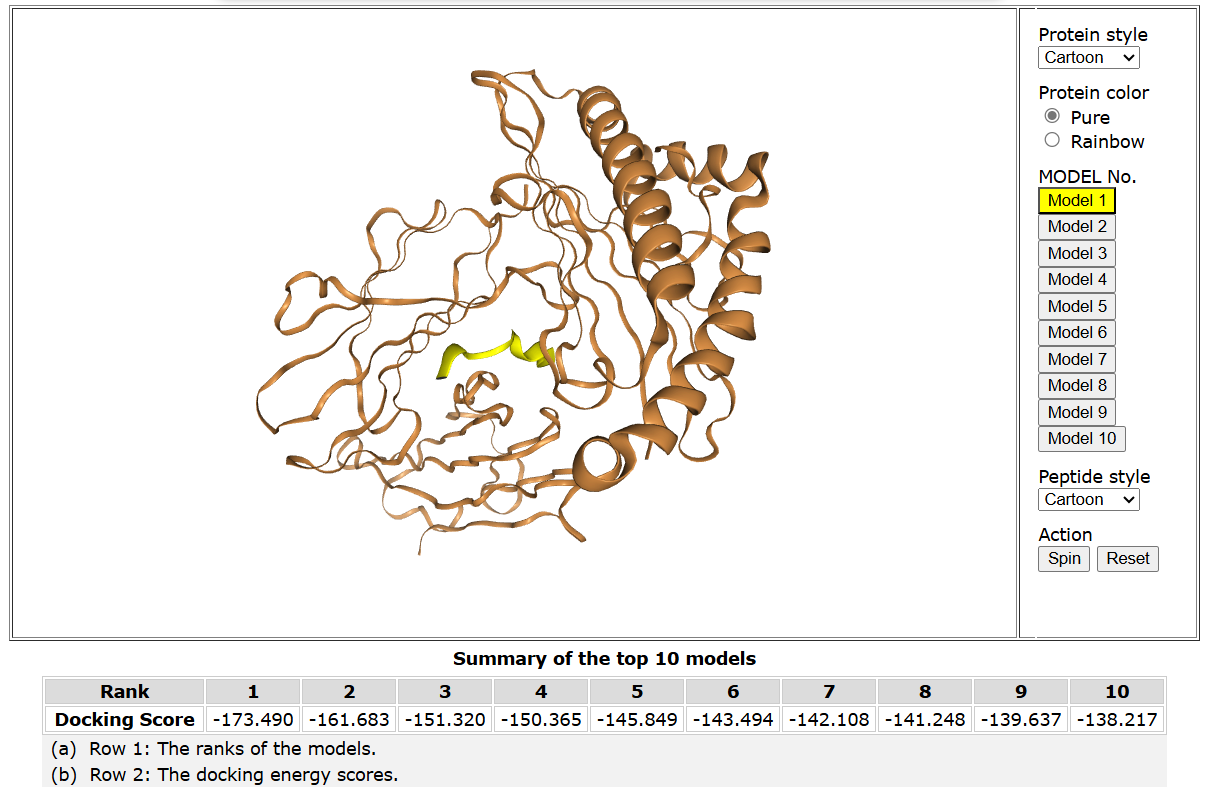


**Supplementary Figure S11:** 3D interaction diagram of AESVPNMSF with HLA A*02:06 (PDB ID 3OXR).


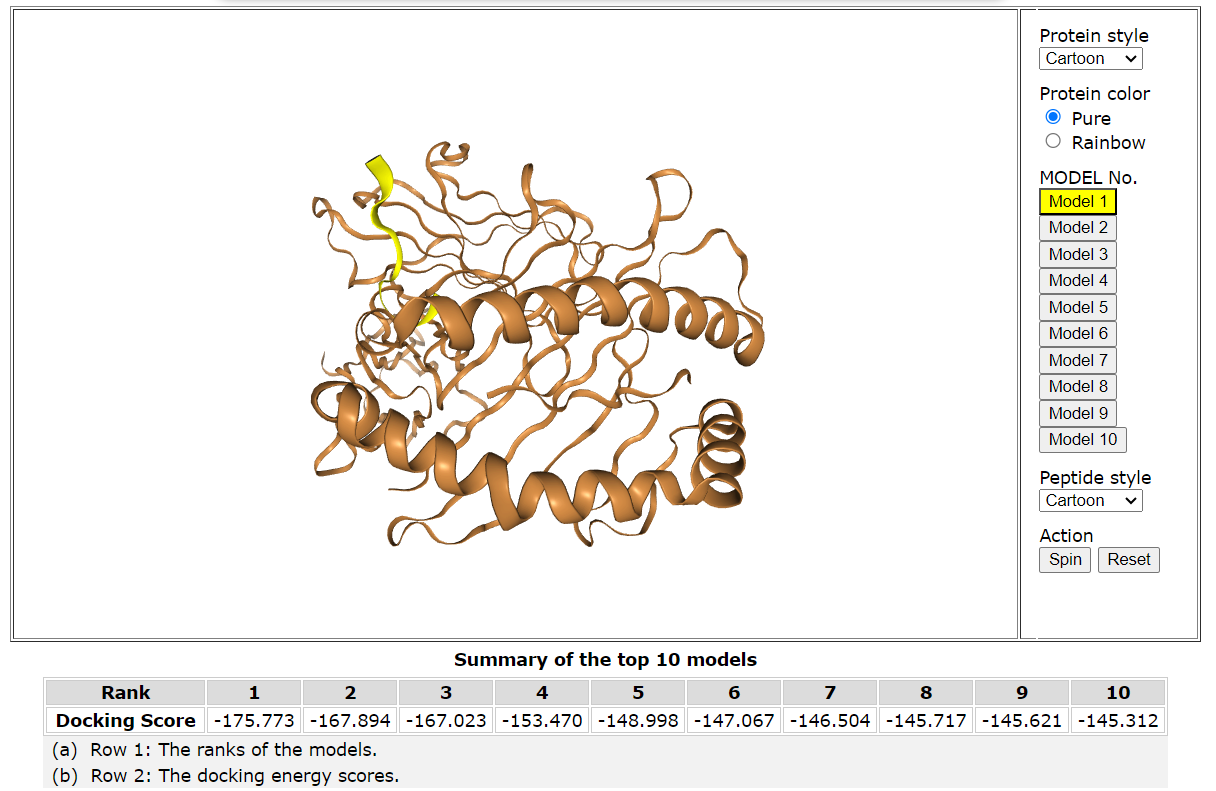


**Supplementary Figure S12:** 3D interaction diagram of AESVPNMSF with HLA-B*40:02 (PDB ID 5IEH).


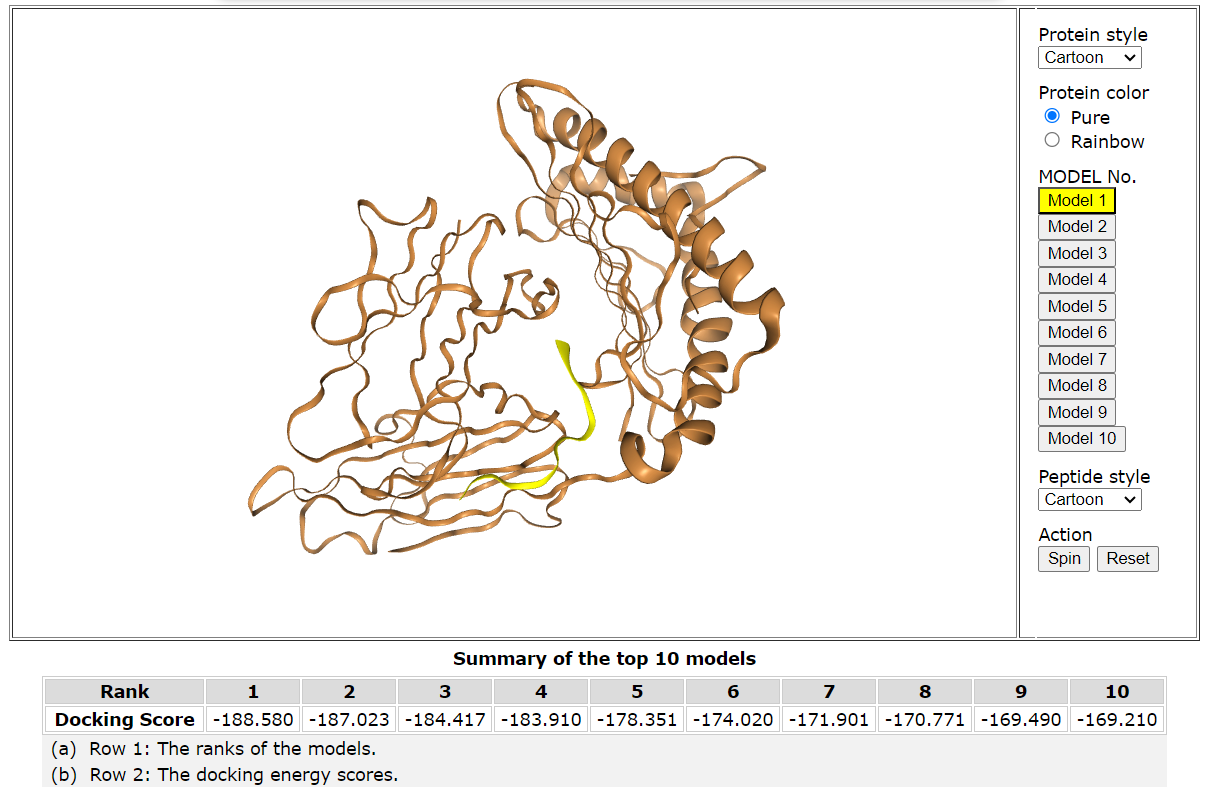


**Supplementary Figure S13:** 3D interaction diagram of LYTDTAFSW with HLA-A*02:01 (PDB ID 4U6Y).


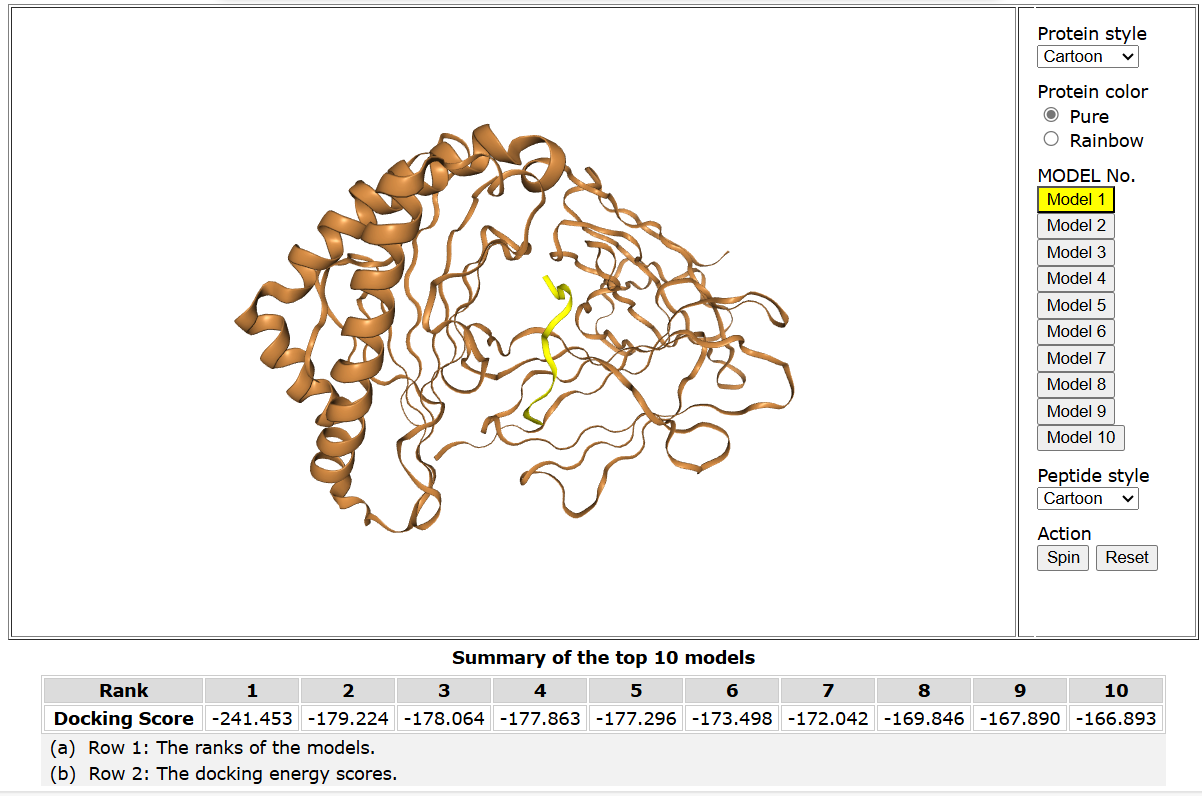


**Supplementary Figure S14:** 3D interaction diagram of LYTDTAFSW with HLA-A*24:02 (PDB ID 8SBK).


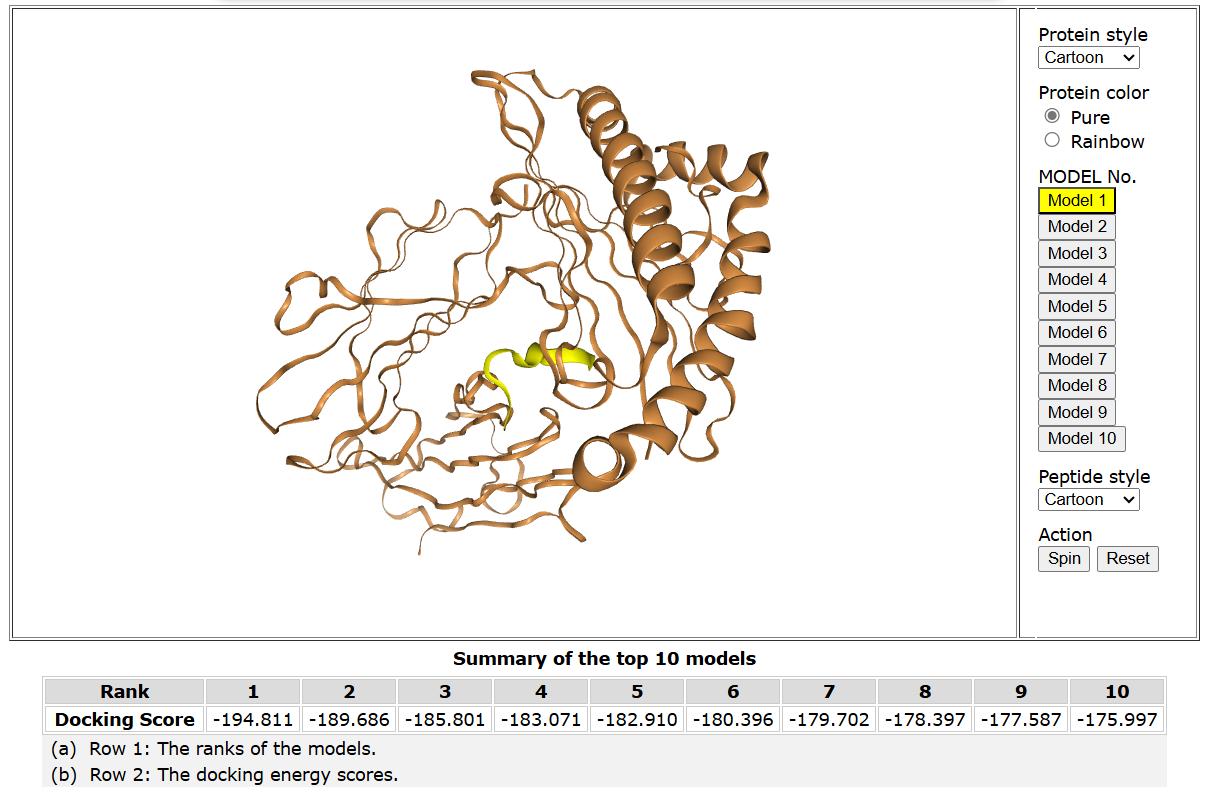


**Supplementary Figure S15:** 3D interaction diagram of LYTDTAFSW with HLA A*02:06 (PDB ID 3OXR).


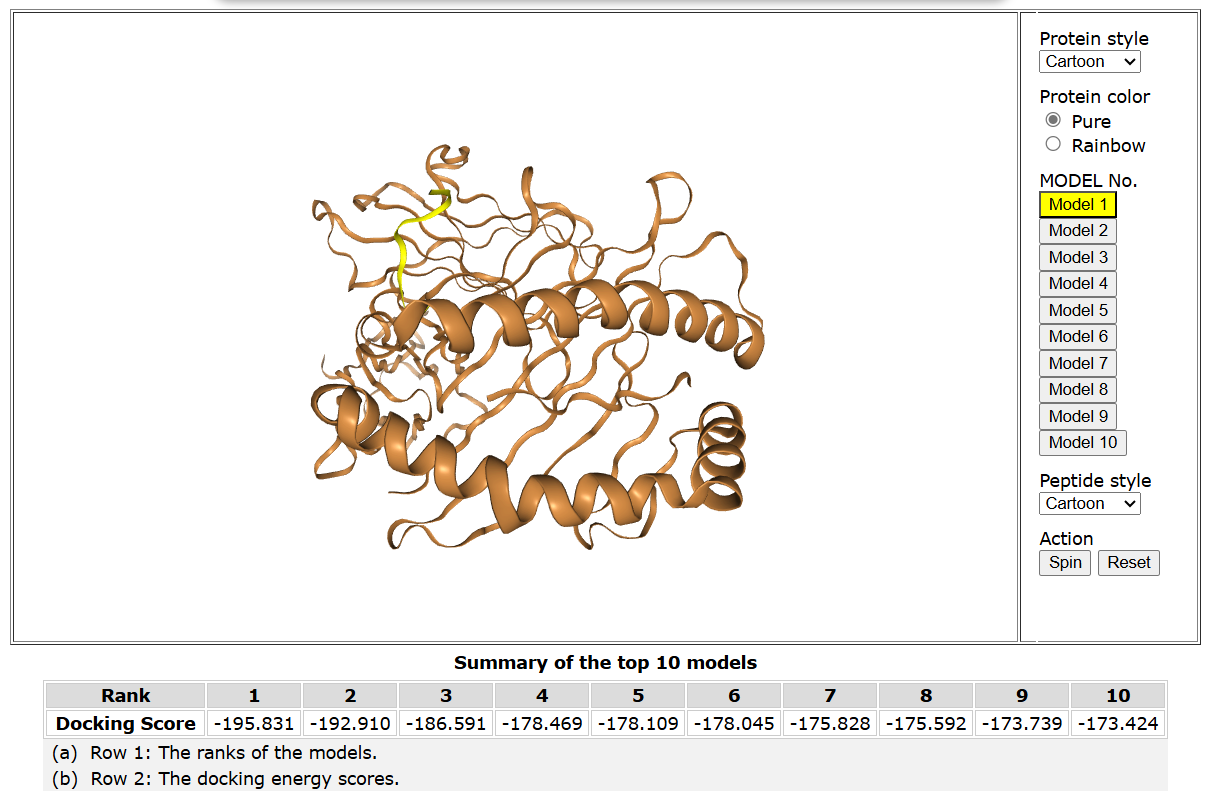


**Supplementary Figure S16:** 3D interaction diagram of LYTDTAFSW with HLA-B*40:02 (PDB ID 5IEH).


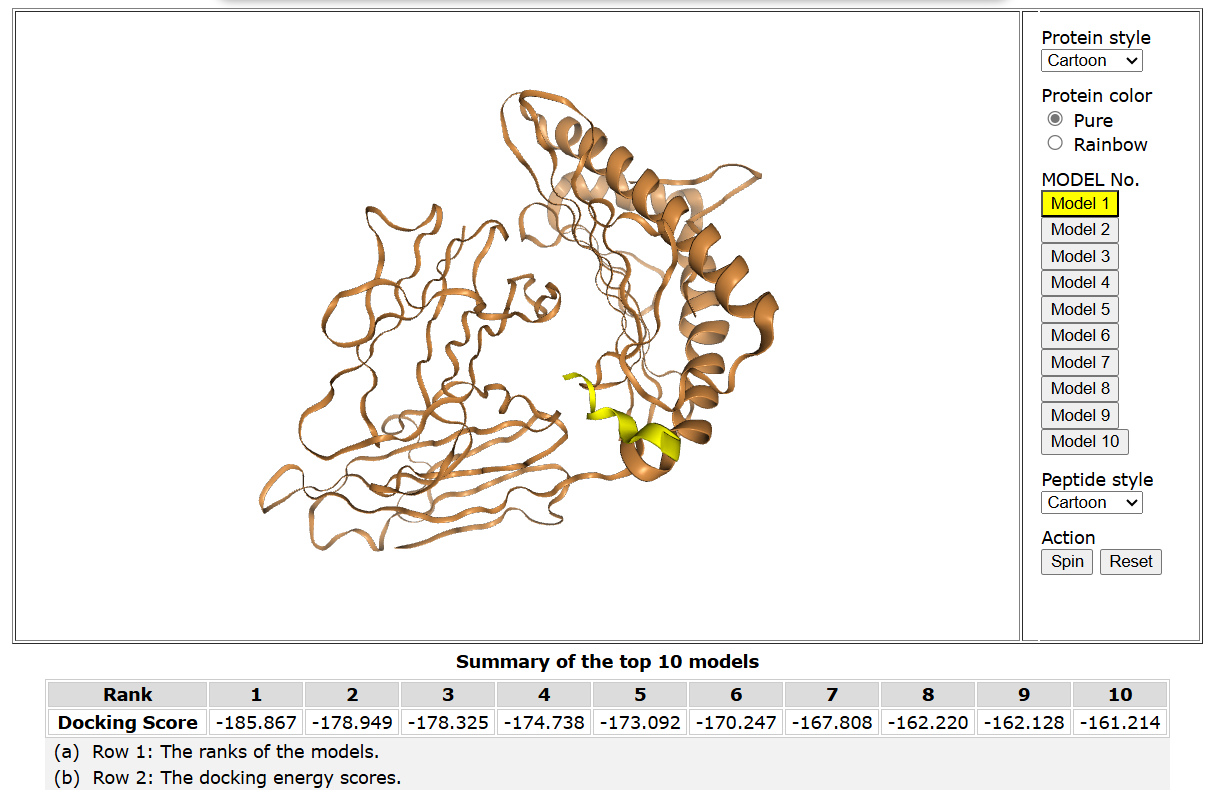


**Supplementary Figure S17:** 3D interaction diagram of FVFDRVLQT with HLA-A*02:01 (PDB ID 4U6Y).


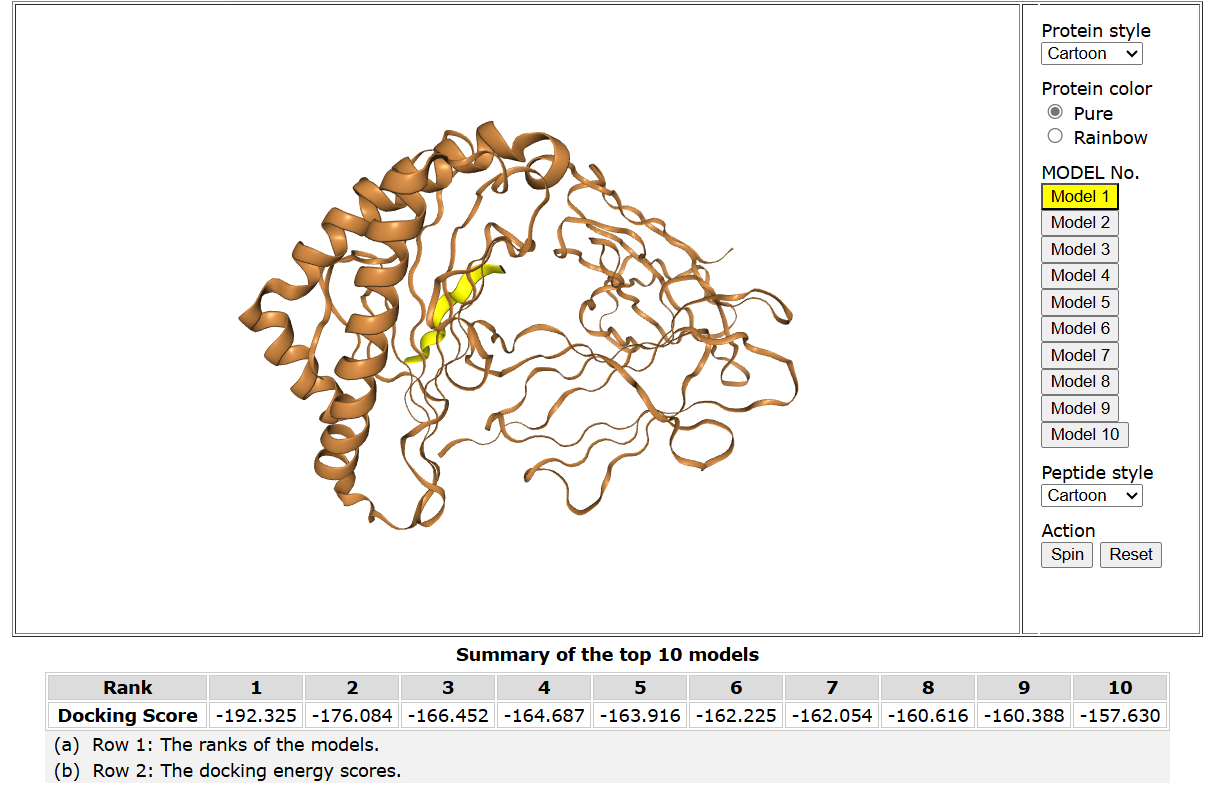


**Supplementary Figure S18:** 3D interaction diagram of FVFDRVLQT with HLA-A*24:02 (PDB ID 8SBK).


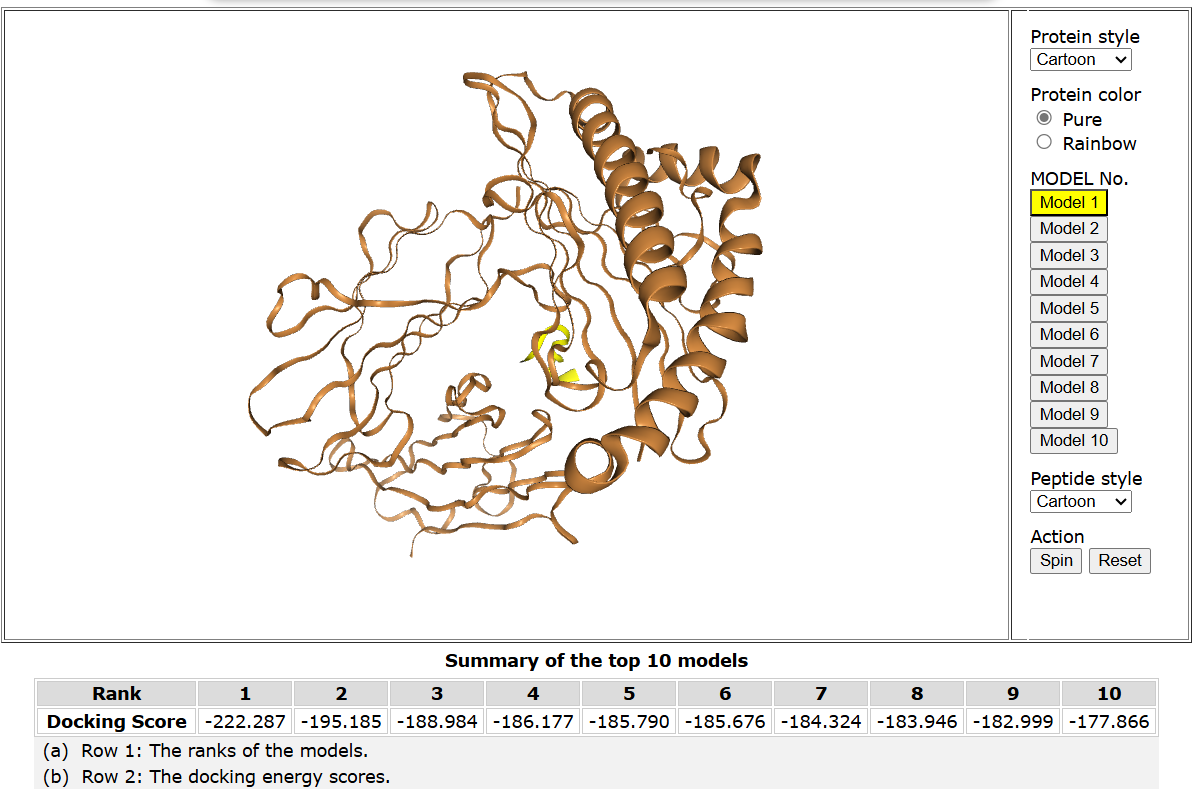


**Supplementary Figure S19:** 3D interaction diagram of FVFDRVLQT with HLA A*02:06 (PDB ID 3OXR).


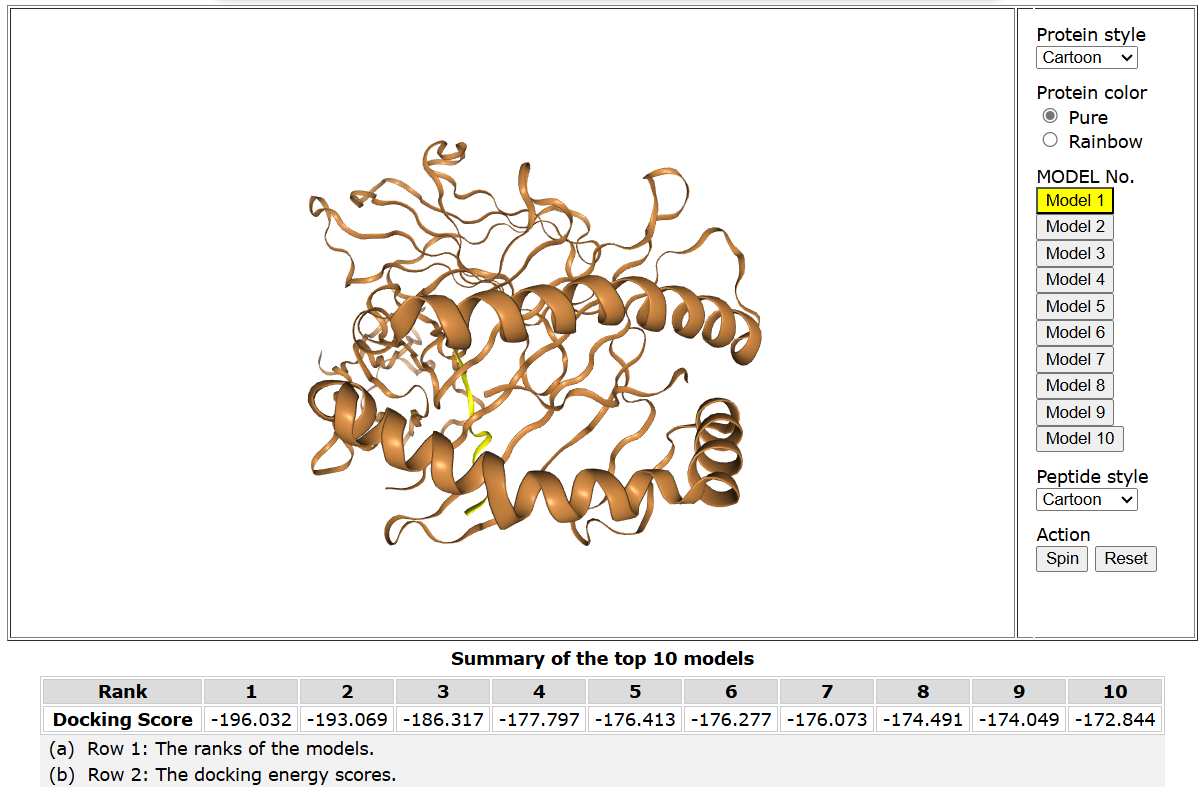


**Supplementary Figure S20:** 3D interaction diagram of FVFDRVLQT with HLA-B*40:02 (PDB ID 5IEH).


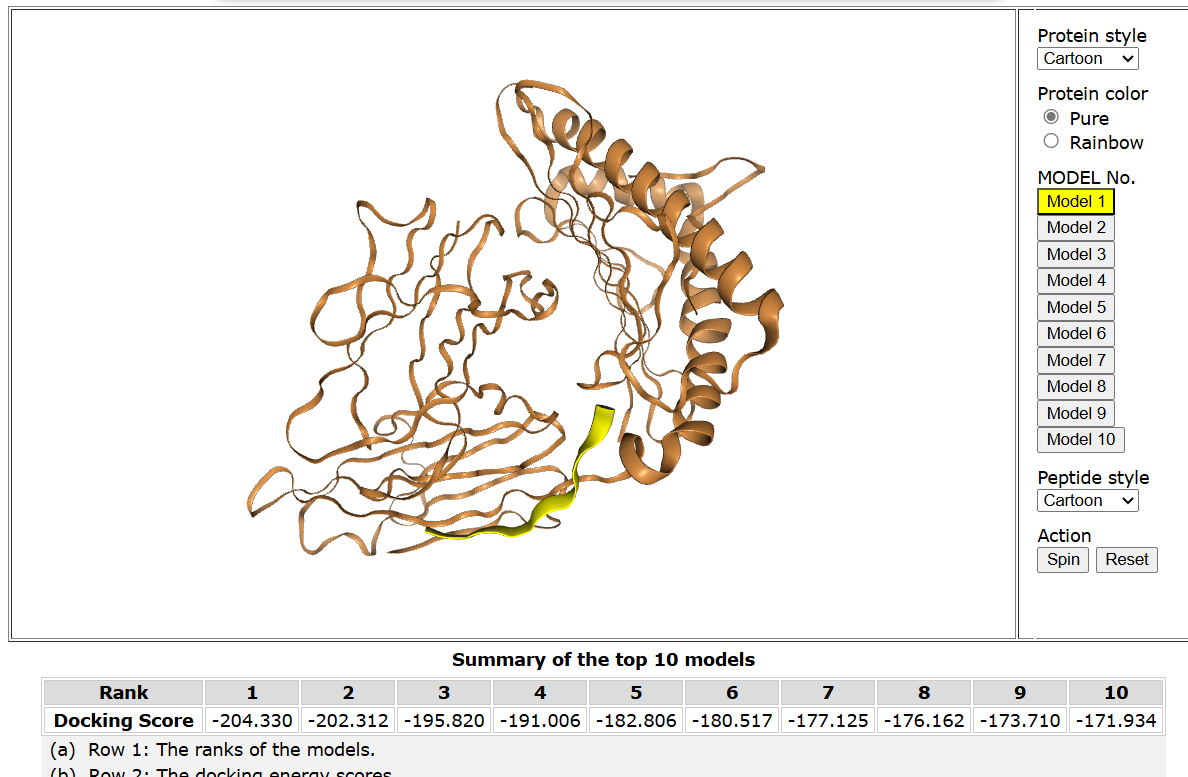


**Supplementary Figure S21:** 3D interaction diagram of LYTDTTFAW with HLA-A*02:01 (PDB ID 4U6Y).


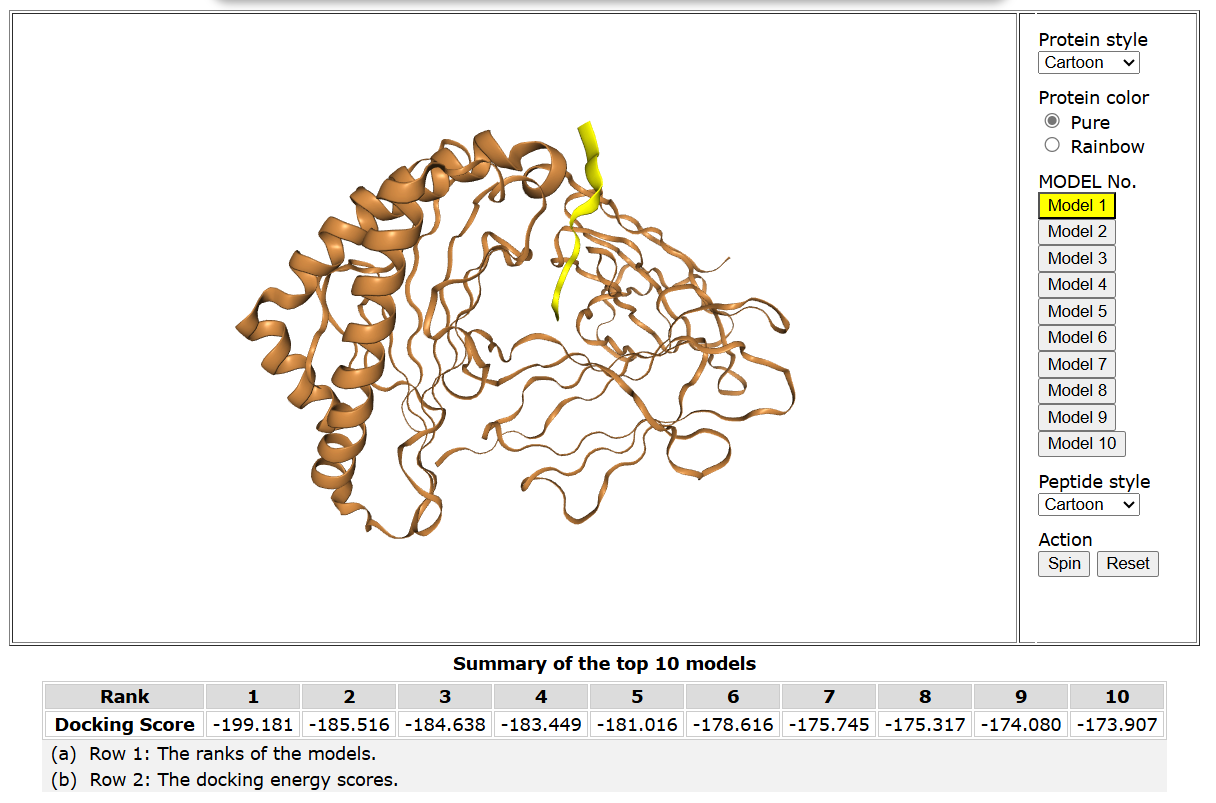


**Supplementary Figure S22:** 3D interaction diagram of LYTDTTFAW with HLA-A*24:02 (PDB ID 8SBK).


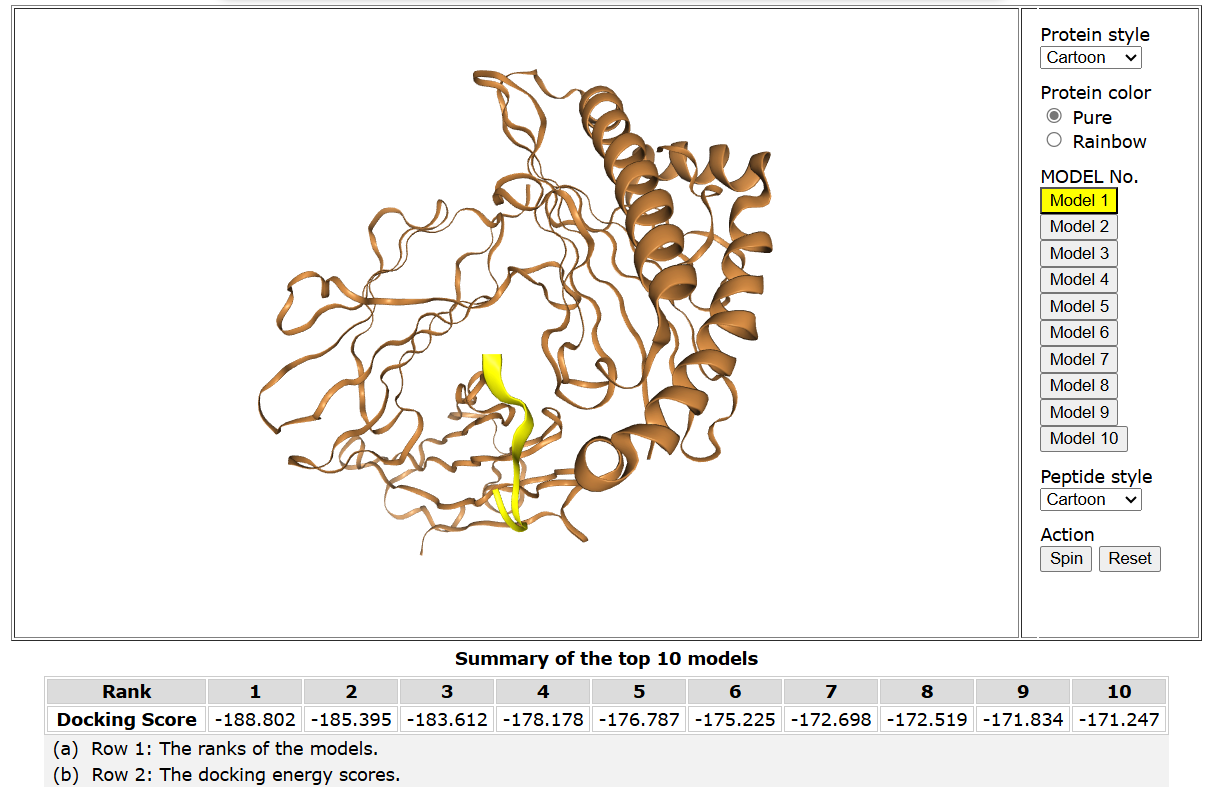


**Supplementary Figure S23:** 3D interaction diagram of LYTDTTFAW with HLA A*02:06 (PDB ID 3OXR).


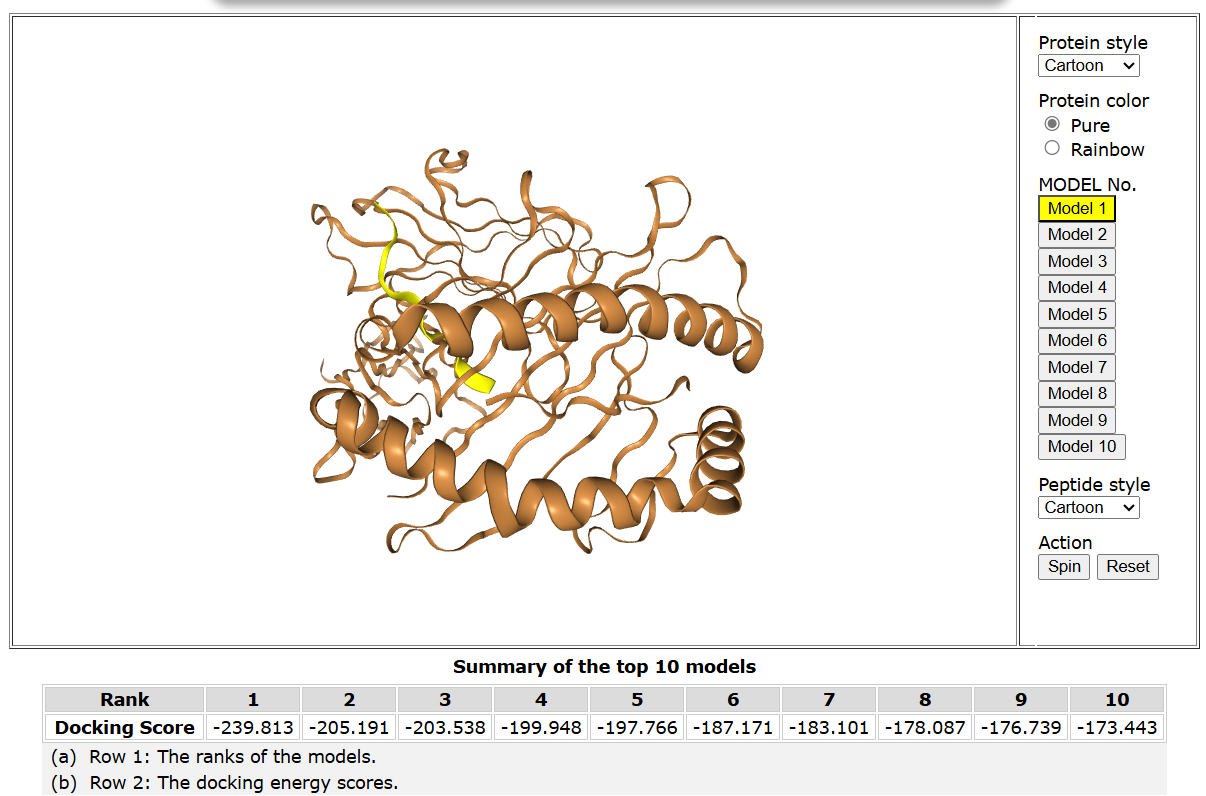


**Supplementary Figure S24:** 3D interaction diagram of LYTDTTFAW with HLA-B*40:02 (PDB ID 5IEH).

**Supplementary Table S2:** Disulfide engineering of the final multiepitope vaccine candidate.

| **Res1 Chain** | **Res1 Seq #** | **Res1 AA** | **Res2 Chain** | **Res2 Seq #** | **Res2 AA** | **Chi3** | **Energy** | **Sum B-Factors** |
| --- | --- | --- | --- | --- | --- | --- | --- | --- |
| A | 4 | LEU | A | 311 | THR | -93.12 | 5.46 | 0 |
| A | 4 | LEU | A | 314 | ALA | -95.05 | 3.25 | 0 |
| A | 25 | VAL | A | 30 | GLU | 104.08 | 5.26 | 0 |
| A | 38 | ALA | A | 40 | VAL | 125.02 | 6.43 | 0 |
| A | 41 | ALA | A | 44 | ALA | 81.68 | 1.15 | 0 |
| A | 48 | ALA | A | 51 | GLY | 96.32 | 1.58 | 0 |
| A | 74 | LYS | A | 321 | ALA | 92.49 | 2.19 | 0 |
| A | 89 | GLY | A | 92 | GLU | 91.83 | 2.76 | 0 |
| A | 95 | ASP | A | 119 | LYS | 99.33 | 5.09 | 0 |
| A | 103 | PRO | A | 104 | LEU | -82.56 | 4.18 | 0 |
| A | 108 | VAL | A | 113 | ALA | 115.51 | 1.71 | 0 |
| A | 109 | ALA | A | 112 | ALA | 112.8 | 6.11 | 0 |
| A | 137 | GLU | A | 141 | SER | 100.46 | 3.06 | 0 |
| A | 137 | GLU | A | 142 | LEU | -87.74 | 3.96 | 0 |
| A | 194 | ALA | A | 324 | THR | -86.52 | 1.2 | 0 |
| A | 227 | MET | A | 289 | PRO | -111.13 | 5.29 | 0 |
| A | 230 | VAL | A | 286 | GLY | 87.69 | 1.97 | 0 |
| A | 282 | GLU | A | 285 | MET | 111.44 | 6.18 | 0 |
| A | 326 | THR | A | 330 | ASN | -96.62 | 5.81 | 0 |
| A | 328 | ARG | A | 415 | PHE | 121.37 | 3.14 | 0 |
| A | 334 | THR | A | 397 | TYR | -108.34 | 2.99 | 0 |
| A | 334 | THR | A | 398 | HIS | 117.61 | 5.88 | 0 |
| A | 340 | PRO | A | 394 | SER | -92.09 | 3.69 | 0 |
| A | 343 | LEU | A | 346 | ARG | 120.77 | 2.27 | 0 |
| A | 347 | GLU | A | 390 | THR | 117.33 | 2.38 | 0 |
| A | 354 | PHE | A | 357 | ASP | 71.61 | 6.2 | 0 |
| A | 357 | ASP | A | 362 | CYS | -106.95 | 4.95 | 0 |
| A | 392 | ASP | A | 395 | ILE | 117.84 | 7.72 | 0 |
| A | 411 | ALA | A | 415 | PHE | 123.61 | 6.51 | 0 |
| A | 416 | ASP | A | 425 | HIS | 109.32 | 2.07 | 0 |
| A | 422 | HIS | A | 425 | HIS | 114.72 | 4.16 | 0 |

**Supplementary Table S3:** Discontinuous B-cell epitopes with their scores predicted by ElliPro.

| **Sl. No.** | **Residue** | **Number of residues** | **Score** |
| --- | --- | --- | --- |
| 1 | A:G335, A:N336, A:A337, A:V338, A:A339, A:P340, A:S341, A:T342, A:L343, A:T344, A:A345, A:R346, A:E347, A:N348, A:P349, A:K350, A:K351, A:E352, A:G353, A:F354, A:G355, A:G356, A:D357, A:P358, A:C359, A:D360, A:P361, A:C362, A:A363, A:T364, A:W365, A:C366, A:D367, A:K368, A:K369, A:E370, A:G371, A:F372, A:G373, A:G374, A:D375, A:P376, A:C377, A:D378, A:P379, A:C380, A:T381, A:T382, A:W383, A:C384, A:D385, A:K386, A:K387, A:T388, A:G389, A:T390, A:K391, A:D392, A:A393, A:S394, A:I395, A:D396, A:Q401, A:A402, A:S403, A:K404, A:K405, A:A406, A:Q407, A:P408 | 70 | 0.802 |
| 2 | A:A133, A:A134, A:K135, A:H136, A:E137, A:W138, A:Q139, A:A140, A:S141, A:L142, A:A143, A:L144, A:A145, A:A146, A:Y147, A:S148, A:L149, A:D150, A:Q151, A:S152, A:V153, A:V154, A:L156, A:A157, A:A158, A:Y159, A:A160, A:E161, A:S162, A:V163, A:A177, A:F178, A:S179, A:W180, A:A181, A:A182 | 36 | 0.69 |
| 3 | A:M1, A:A2, A:K3, A:L4, A:S5, A:D7, A:E8, A:D11, A:A12, A:E15, A:M16, A:L19, A:E20, A:D23, A:K27, A:F28, A:M227, A:Q228, A:I229, A:V230, A:S231, A:L232, A:Q233, A:L234, A:N235, A:K236, A:M237, A:K238, A:S239, A:R240, A:A241, A:A242, A:Y243, A:D244, A:T245, A:M246, A:Q247, A:I248, A:V249, A:S250, A:L251, A:Q252, A:L253, A:N254, A:K255, A:M256, A:K257, A:S258, A:A259, A:A260, A:Y261, A:P270, A:K271, A:S272, A:A275, A:I276, A:A278, A:Y279, A:N280, A:K281, A:E282, A:F283, A:Q284, A:M285, A:G286, A:A287, A:K288, A:P289, A:T290, A:A291, A:T292, A:T293, A:G294, A:A295, A:A296, A:A298, A:D299, A:T300, A:I301, A:R302, A:I303, A:A304, A:Q305, A:P306, A:K307, A:S308, A:A309, A:T310, A:T311, A:V312, A:A313, A:A314, A:T316, A:G317, A:N318, A:A319, A:T320 | 97 | 0.683 |
| 4 | A:A52, A:E55, A:A56, A:A57, A:E59, A:Q60, A:S61, A:E62, A:F63, A:I84, A:V85, A:S86, A:G87, A:L88 | 14 | 0.613 |
| 5 | A:V108, A:A109, A:K110, A:E111 | 4 | 0.553 |
| 6 | A:D416, A:V417, A:T418, A:T419, A:L420, A:N421, A:H422, A:H423, A:H424, A:H425 | 10 | 0.503 |


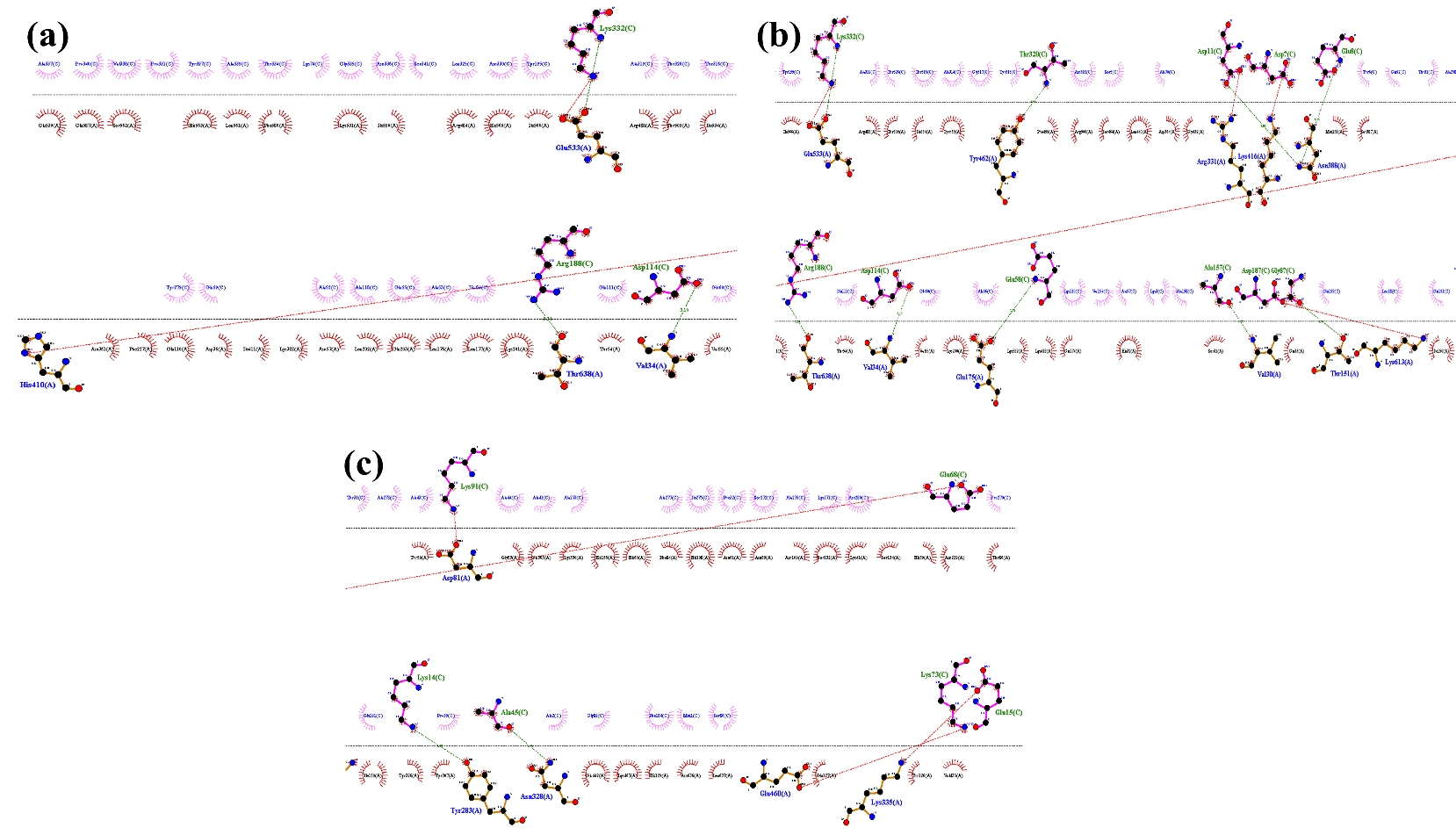
**Supplementary Figure S25:** 2D-interaction diagram (Zoom form) of the protein-protein complex generated by the GRAMM-X server where chain A is from the TLR-3 receptor and chain C is the vaccine construct.


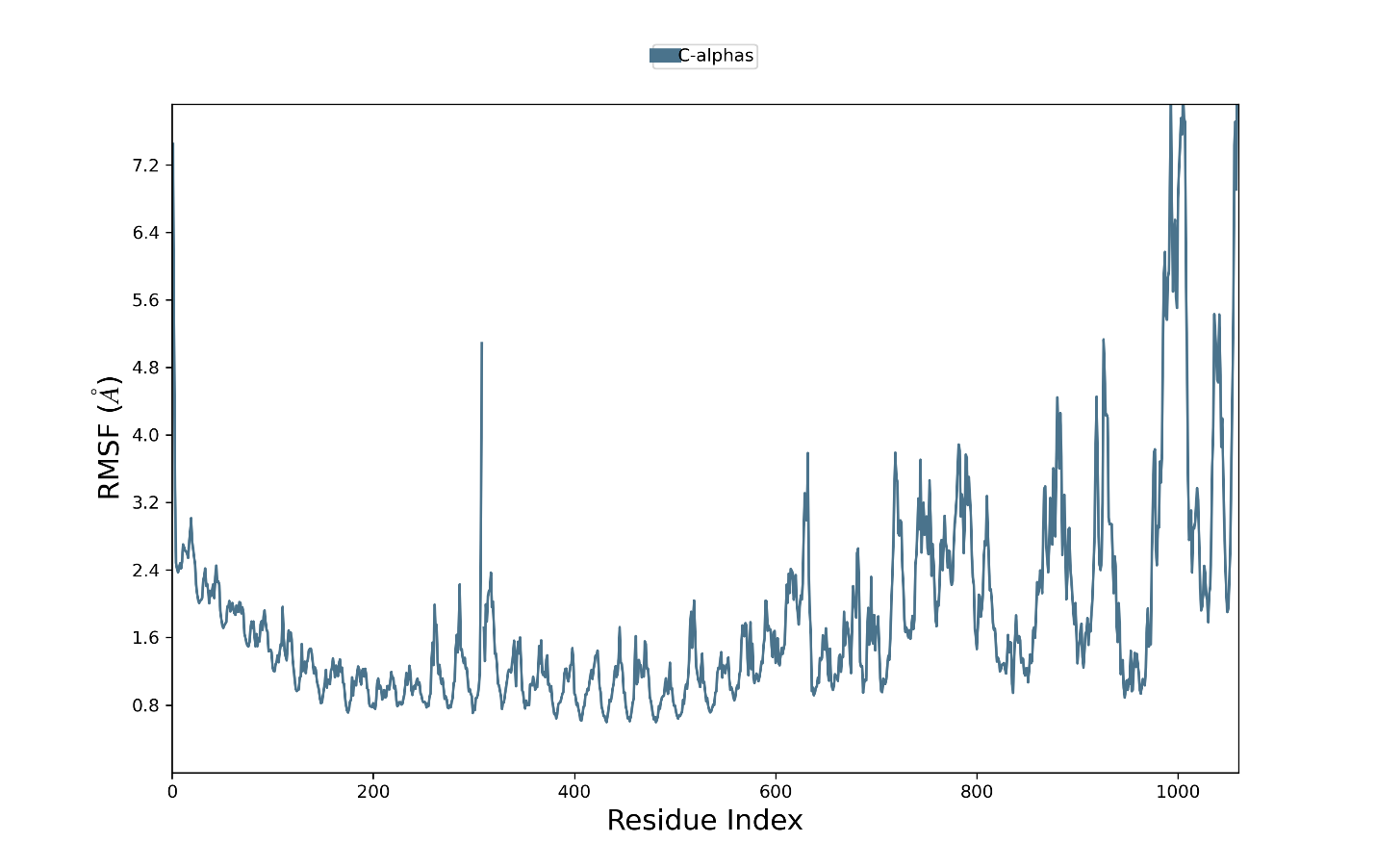


**Supplementary Figure S26:** RMSF plot of the vaccine-TLR3 complex.


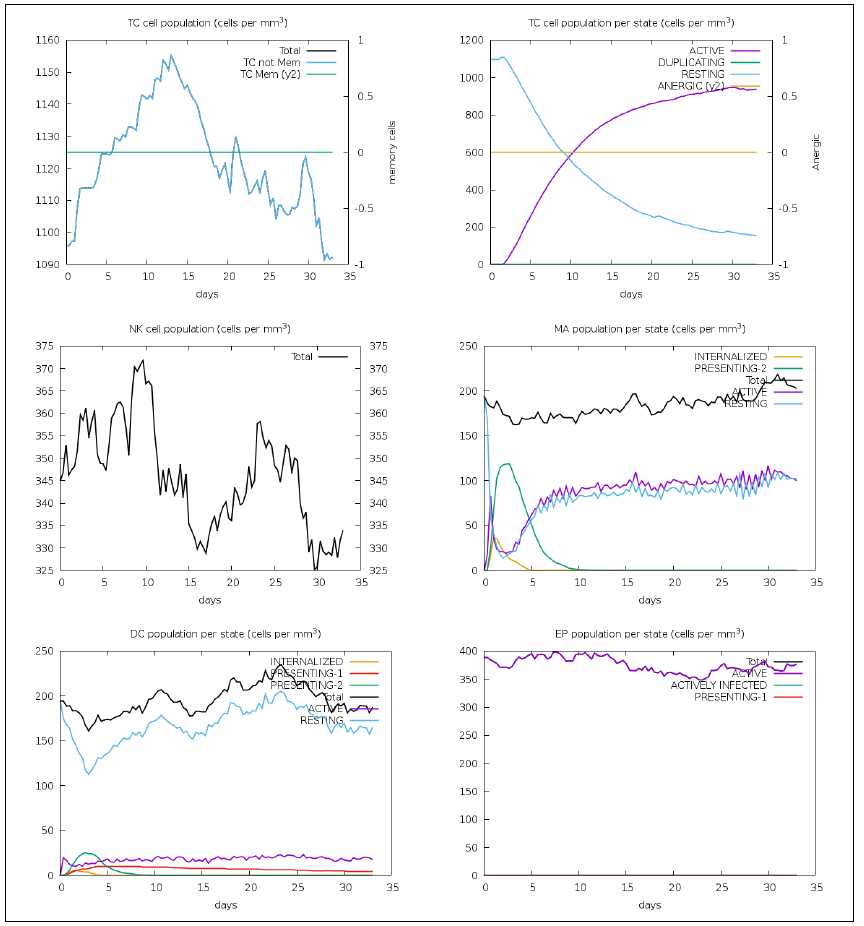


**Supplementary Figure S27:** Immune cells count: DC, dendritic cells; EP, eosinophil; NK, natural killer cells; TC, cytotoxic T-cells.


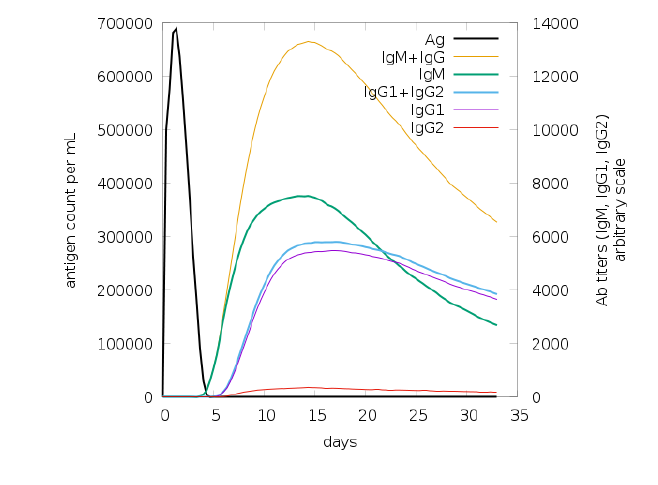


**Supplementary Figure S28:** The virus, the immunoglobulins, and the immunocomplexes.


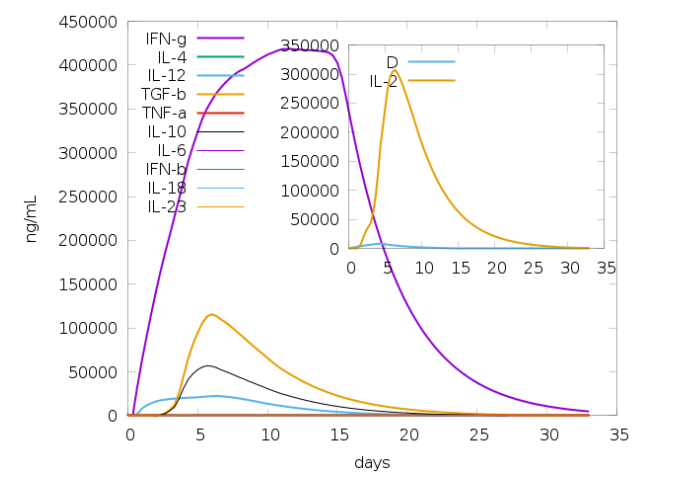


**Supplementary Figure S29:** Concentration of cytokines and interleukins. Inset plot shows danger signal together with leukocyte growth factor IL-2.
